# Th17 cell master transcription factor RORC2 regulates HIV-1 gene expression and viral outgrowth

**DOI:** 10.1073/pnas.2105927118

**Published:** 2021-11-24

**Authors:** Tomas Raul Wiche Salinas, Yuwei Zhang, Daniele Sarnello, Alexander Zhyvoloup, Laurence Raymond Marchand, Augustine Fert, Delphine Planas, Manivel Lodha, Debashree Chatterjee, Katarzyna Karwacz, Sally Oxenford, Jean-Pierre Routy, David Irlbeck, Heather Amrine-Madsen, Petronela Ancuta, Ariberto Fassati

**Affiliations:** ^a^Centre de recherche du Centre Hospitalier de l'Université de Montréal, Université de Montréal, Montréal, QC H2X 0A9, Canada;; ^b^Département de Microbiologie, Infectiologie et Immunologie, Faculté de Médecine, Université de Montréal, Montréal, QC H3T 1J4, Canada;; ^c^Institute of Immunity and Transplantation, University College London, London NW3 2QG, United Kingdom;; ^d^Translational Research Office—Medicinal Chemistry, University College London School of Pharmacy, London WC1N 1AX, United Kingdom;; ^e^Division of Hematology and Chronic Viral Illness Service, McGill University Medical Centre, Montréal, QC H4A 3J1, Canada;; ^f^ViiV Healthcare, Research Triangle Park, NC 27709-3398;; ^g^Division of Infection and Immunity, University College London, London WC1E 6JF, United Kingdom

**Keywords:** HIV-1, RORC2, Th17, gene expression, hormone receptor

## Abstract

Among CD4^+^ T cells, T helper 17 (Th17) cells are particularly susceptible to HIV-1 infection and are depleted from mucosal sites, which causes damage to the gut barrier, resulting in a microbial translocation-induced systemic inflammation, a hallmark of disease progression. Furthermore, a proportion of latently infected Th17 cells persist long term in the gastrointestinal lymphatic tract where a low-level HIV-1 transcription is observed. This residual viremia contributes to chronic immune activation. Thus, Th17 cells are key players in HIV pathogenesis and viral persistence. It is, however, unclear why these cells are highly susceptible to HIV-1 infection. Th17 cell differentiation depends on the expression of the master transcriptional regulator *RORC2*, *a* retinoic acid-related nuclear hormone receptor that regulates specific transcriptional programs by binding to promoter/enhancer DNA. Here, we report that RORC2 is a key host cofactor for HIV replication in Th17 cells. We found that specific inhibitors that bind to the RORC2 ligand-binding domain reduced HIV replication in CD4^+^ T cells. The depletion of RORC2 inhibited HIV-1 infection, whereas its overexpression enhanced it. RORC2 was also found to promote HIV-1 gene expression by binding to the nuclear receptor responsive element in the HIV-1 long terminal repeats (LTR). In treated HIV-1 patients, RORC2^+^ CD4 T cells contained more proviral DNA than RORC2^−^ cells. Pharmacological inhibition of RORC2 potently reduced HIV-1 outgrowth in CD4^+^ T cells from antiretroviral-treated patients. Altogether, these results provide an explanation as to why Th17 cells are highly susceptible to HIV-1 infection and suggest that RORC2 may be a cell-specific target for HIV-1 therapy.

## Significance

HIV-1 infects CD4 T cells, and, among these, T helper 17 (Th17) cells are known to be particularly permissive for virus replication. The infection of Th17 cells is critical for AIDS pathogenesis and viral persistence. It is, however, not clear why these cells are highly permissive to HIV-1. We found that Th17 cell permissiveness depends on the expression of the hormone receptor RORC2, which is the master transcriptional regulator of Th17 cell differentiation. We identify RORC2 as a cell-specific host-dependency factor that can be targeted by small molecules. Our results suggest that RORC2 may be a cell-specific target to mitigate the loss of Th17 cells as a consequence of their preferential HIV-1 infection.

A hallmark of HIV-1 infection is systemic inflammation, which can best predict disease progression (1, 2). A significant proportion of people living with HIV (PLWH) with undetectable plasma viral load during antiretroviral therapy (ART) have systemic inflammation, the severity of which correlates with the overall mortality, morbidity, and comorbidity (1, 2). This inflammation also supports viral persistence by promoting homeostatic proliferation and clonal expansion of memory CD4^+^ T cells carrying HIV-1 reservoirs (3, 4) by enhancing their migration into lymphatic organs and by inducing their activation, which stimulates local HIV-1 infection and reactivation from latency (5). The systemic inflammation observed in some virally suppressed individuals indicates that the widespread and ongoing virus replication is not the main cause of this immune disorder; instead, substantial evidence suggests that inflammation has a predominantly indirect origin and is linked to alterations at mucosal sites (1, 6, 7).

T helper 17 (Th17) cells are a heterogeneous subset of CD4^+^ T cells that express the chemokine receptor CCR6 and produce lineage-specific cytokines such as IL-17A, IL-21, and IL-22 (8–10). They are mainly found in the intestinal lamina propria and vaginal cervix mucosa, where they maintain the immunological barrier to microbiota including bacteria and fungi (9–11). Remarkably, Th17 cells, which are preferentially targeted by HIV-1 in vitro and in vivo (10, 11), are also among the very first cells infected upon Simian immunodeficiency virus (SIV) vaginal exposure in macaques (12). As a consequence, Th17 cells are depleted from the gut and vaginal mucosa during acute infection in both PLWH (10, 11) and in SIV-infected monkeys (13, 14). The depletion of Th17 cells from the gastrointestinal lymphatic tract (GALT) of HIV-1–infected individuals has critical consequences for disease progression and viral persistence (7). Studies in PLWH and in pathogenic models of SIV infection showed that the loss of Th17 cells correlates with systemic inflammation, putatively via the disruption of the immunological homeostasis at the mucosal barriers and the translocation of bacterial products from the mucosa into the circulation (15, 16). Bacterial and fungal products then trigger the release of proinflammatory cytokines by various immune cells, establishing and maintaining systemic inflammation (7, 16, 17). These events are so critical that the loss or maintenance of Th17 cells can discriminate between pathogenic and nonpathogenic lentiviral infections (13, 17). Nevertheless, a proportion of infected Th17 cells are long lived and constitute a component of the viral reservoir in the gut (8, 18–22), underscoring their importance in maintaining viral persistence during ART.

Despite these major insights, we do not fully understand why Th17 cells are preferentially targeted by HIV-1 for infection and how they are lost during the early acute phases of the disease (10, 11). Also, the mechanisms that govern HIV-1 reactivation from latency in long-lived Th17 cells carrying viral reservoirs in ART-treated PLWH are still unclear.

The differentiation and effector functions of Th17 cells depend on the expression of the master regulator retinoic acid receptor-related orphan receptor 2, RORC2 (RORγt in mice) (23–25). Here, we report that RORC2 is a bona fide host cofactor for HIV-1 gene expression in Th17 cells and identify small molecule RORC2 inhibitors that potently block HIV-1 replication/outgrowth without widespread cell toxicity. These findings establish a key molecular link between RORC2 expression and HIV-1 replication and suggest that RORC2 may be a Th17 cell-specific target to mitigate the loss of Th17 cells induced by their preferential HIV-1 infection.

## Results

### RORC2 Is a Druggable Host Dependency Factor for HIV-1.

**Fig. 1.**
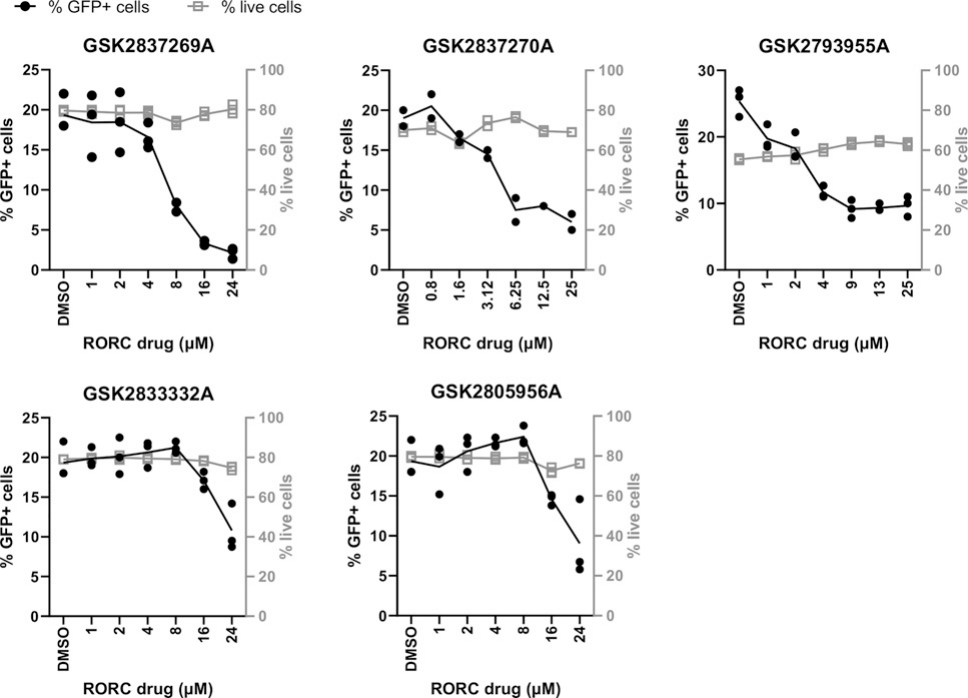
RORC inhibitors inhibit HIV-1 infection. Jurkat cells were infected with single-cycle, VSV-G–pseudotyped HIV-1 LAI_GFP_ at an MOI of 0.2 in the presence of the indicated concentrations of RORC2 inhibitors. The cells were analyzed 48 h postinfection by flow cytometry to measure the percentage of GFP+ cells. The proportion of live cells was simultaneously assessed by AlamaR blue, *n* = 3.

In a previous compound screening experiment to find small molecules with anti-HIV-1 activity, we identified two hits (digoxin and digitoxin) that are known antagonists of both RORC2 and the Na^+^/K^+^ ATPase (26–28). Although we and others showed that digoxin affects HIV-1 gene expression mainly via the Na^+^/K^+^ ATPase (27–29), digoxin-mediated inhibition of HIV-1 infection was also observed in Jurkat cells expressing the mouse Na^+^/K^+^ ATPase (27), which does not bind to digoxin, suggesting that RORC2 might be a secondary target. To confirm and extend our initial observation, we tested several new well-characterized RORC2 inhibitors developed by GlaxoSmithKline (GSK). Compounds GSK261805A, GSK2837270A, GSK2793955A, and GSK283726 are bound to the RORC2 ligand-binding domain (LBD) with nanomolar affinity and displaced steroid receptor coactivator-1 (SRC1) (30), whereas GSK2833332A and GSK2805956A had no measurable affinity up to 10 µM (*SI Appendix*, Table 1). The compounds with a high affinity for RORC2 LBD also inhibited expression of a reporter luciferase gene under the control of the human IL-17A enhancer/promoter with micromolar IC50s (*SI Appendix*, Table 1), which goes in agreement with the observation that RORC2 induces IL-17 expression by binding at promoter and enhancer regions in the IL-17 locus (31). Initially, we sought to test the compounds’ antiviral activity in Jurkat cells, which does not require stimulation and whose susceptibility to HIV-1 infection is less variable than that of primary CD4^+^ T cells. To determine if Jurkat cells express RORC2 and hence may be sensitive to the compounds, we performed RT-qPCR using two sets of primers that specifically amplify either RORC2 or RORC1, an alternative spliced isoform that differs from RORC2 at its N-terminal region and is not normally expressed in CD4^+^ T cells (32). This analysis showed that Jurkat cells and memory CD4^+^ T cells express RORC2 but not RORC1 transcripts (*SI Appendix*, Fig. 1 *A* and *B*); RORC2 protein expression was also confirmed in Jurkat cells by Western blot (*SI Appendix*, Fig. 1*C*). A single cycle HIV-1 LAIΔenv virus expressing green fluorescent protein (GFP) and pseudotyped with vesicular stomatitis virus G-gycoprotein (hereafter called HIV-1 LAI_GFP_) (33) was used to infect Jurkat cells in the presence of increasing concentrations of RORC2 inhibitors or dimethyl sulfoxide (DMSO). Infected cells were analyzed by flow cytometry 48 h postinfection and assessed for cell toxicity by alamarBlue or LIVE/DEAD staining. Compound GSK2837269A was the most potent followed by GSK2837270A and GSK2793955A, whereas GSK2805956A and GSK2833332A showed lower potency (Fig. 1), which is consistent with their low affinity for RORC2 (*SI Appendix*, Table 1). None of the compounds showed detectable cytotoxicity.

**Fig. 2.**
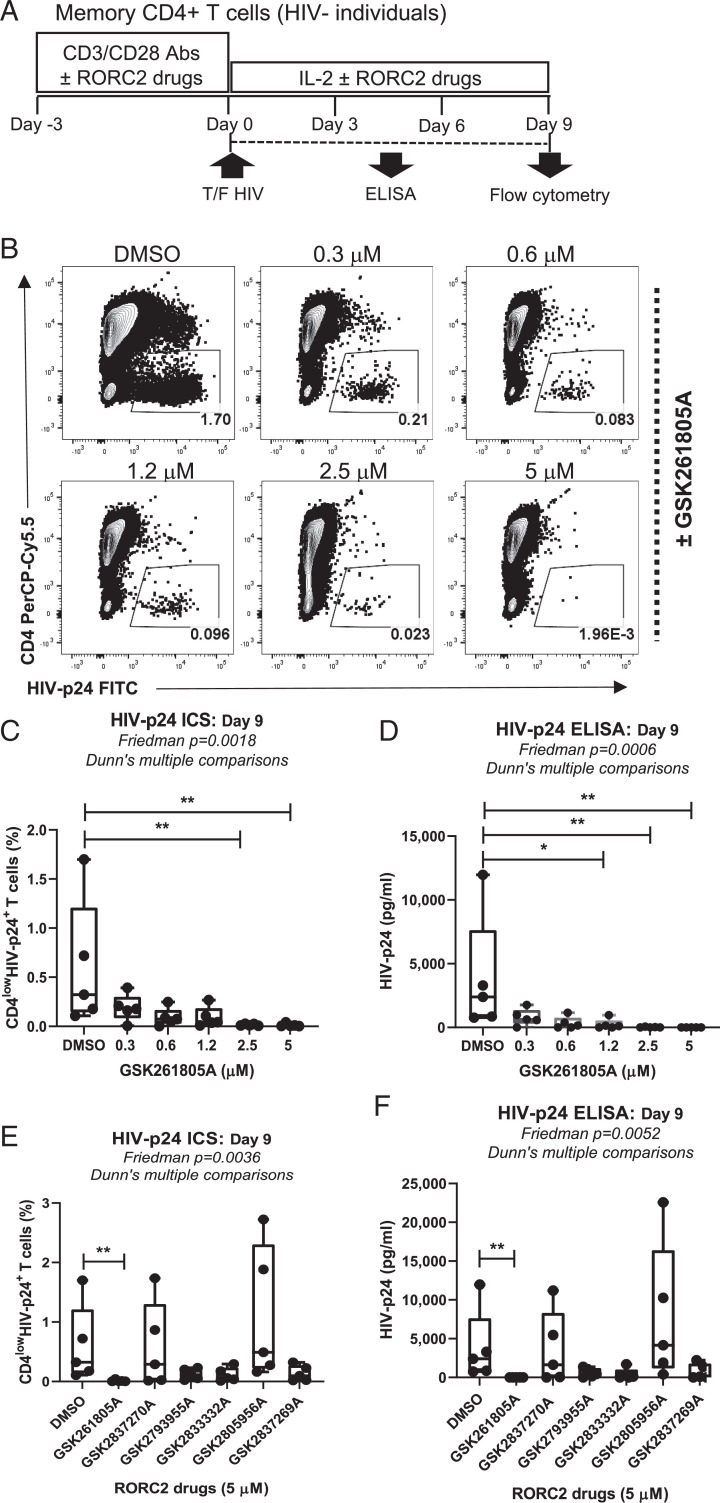
RORC inhibitors reduce HIV-1 replication in primary CD4^+^ T cells in vitro as shown in the experimental flowchart. (*A*) Memory CD4^+^ T cells isolated from *n* = 5 healthy donors were stimulated with CD3/CD28 antibodies in the presence or absence of the indicated concentrations of RORC2 inhibitors for 3 d. Then, cells were exposed to HIV_THRO_ for 3 h. HIV-infected CD4^+^ memory T cells were cultured with IL-2 in the presence of the indicated concentrations of RORC2 inhibitors for 9 d. The media was refreshed with IL-2 and RORC2 inhibitors every 3 d. (*B*) The intracellular expression of HIV-p24 in CD4^+^ memory T cells was quantified by flow cytometry after staining with fluorochrome-conjugated CD4 and HIV-p24 Abs at day 9 postinfection. The contour plots of the frequency of CD4^low^HIV-p24^+^ T cells in one representative donor are shown. (*C*) The statistical analysis of the frequency of CD4^low^HIV-p24^+^ T cells within the total memory CD4^+^ T cells exposed to the indicated concentration of GSK261805A from *n* = 5 donors 9 d postinfection (*D*) HIV-p24 levels measured in cell-culture supernatants by ELISA at day 9 postinfection. (*E*) The statistical analysis of the frequency of CD4^low^HIV-p24^+^ T cells within the memory subset and (*F*) HIV-p24 levels in the cell-culture supernatant in the presence of the indicated RORC2 inhibitors (5 μM). Friedman *P* values, with Dunn’s multiple comparison significance, are indicated on the graphs.

**Fig. 3.**
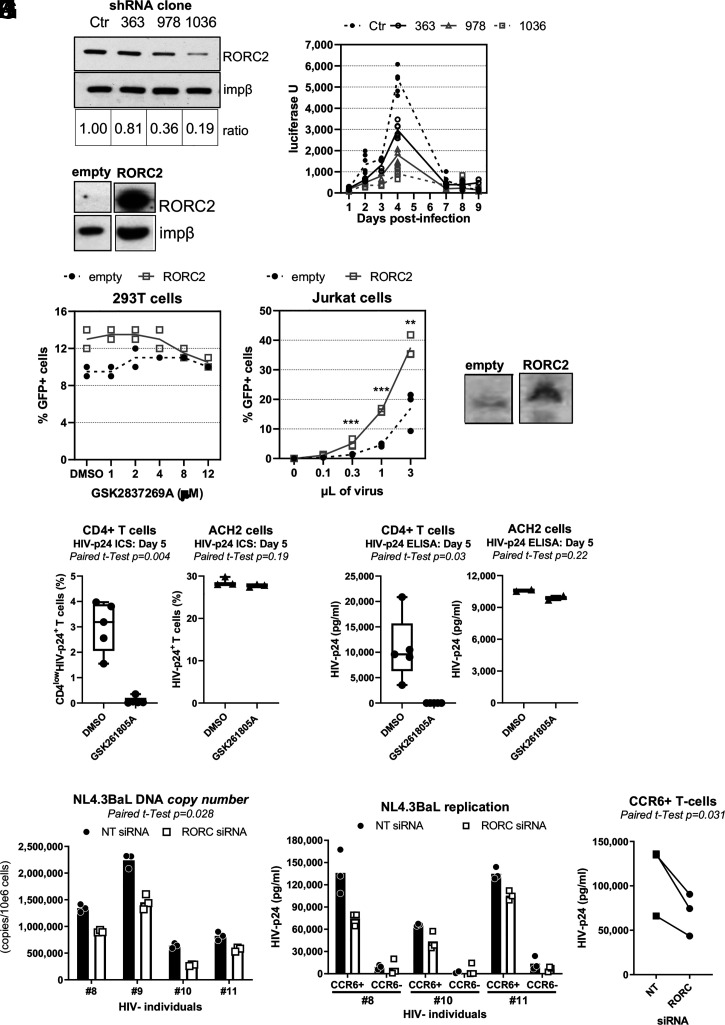
RORC2 is a host cofactor for HIV-1 infection of CD4^+^ T cells. (*A*) Luciferase Jurkat 1G5 indicator cells were transduced with three different shRNAs that target RORC2 or a nontargeting shRNA (Ctr) and selected in puromycin. RORC2 protein levels were analyzed by Western blot, and impβ was used as a loading control. The signal was quantified by ImageJ, and the ratio of RORC2 versus impβ is indicated in *Lower*. (*B*) The 1G5 cells transduced with the same shRNAs were infected with HIV-1_NL4.3_, and the luciferase signal was measured at the indicated time points. (*C*) An EV plasmid or the myc-RORC2 cDNA was transfected into 293T cells. After selection in puromycin, the cells were analyzed by Western blot. (*D*) The same 293T cells were infected with single-cycle HIV-1 LAI_GFP_ at an MOI of 0.1 in the presence of the indicated concentrations of GSK2837269A, and 40 h later, the cells were analyzed by flow cytometry to measure the percentage of GFP+ cells (*n* = 2). (*E*) Jurkat cells were transduced with a retroviral vector expressing myc-RORC2 or an EV, selected in media containing blasticidin for 7 d, and infected with the indicated volumes of single-cycle HIV-1 LAI_GFP_ (10^7^ i.u./mL). The cells were analyzed 40 h after infection by flow cytometry to measure the percentage of GFP+ cells. ***P < 0.005, **P = 0.008 two tailed Student’s *t* test, *n* = 3. (*F*) The expression of RORC2 in these cells was confirmed by Western blot. (*G* and *H*) Memory CD4^+^ T cells isolated from *n* = 5 HIV− participants and ACH2 cells were cultured in parallel using the experimental design depicted in Fig. 2*A*. CD3/CD28-activated CD4^+^ T cells exposed to HIV_THRO_ for 3 h, and ACH2 cells were cultured with IL-2 in the presence or the absence of GSK261805A (5 μM) for 5 d. (*G*) The intracellular expression of HIV-p24 was quantified by flow cytometry upon staining with fluorochrome-conjugated CD4 and HIV-p24 Abs. (*H*) HIV-p24 levels in cell-culture supernatants were measured by ELISA. The paired Student’s *t* test *P* values are indicated on the graphs. (*I*–*K*) CD3/CD28-activated total memory CD4^+^ T cells from *n* = 4 HIV− donors (*I*) or FACS-sorted memory CCR6^+^ and CCR6^−^ CD4^+^ T cells from *n* = 3 HIV− donors (*J* and *K*) were nucleofected with Dharmacon on target smart siRNA pools specific for RORC2 PI Tor a nontargeting siRNA using the Amaxa technology. The cells were then exposed to HIV_NL4.3BaL_ (50 ng per 10^6^ cells), and HIV-DNA integration was quantified by nested real-time PCR at day 3 postinfection. HIV-DNA integration in memory CD4^+^ T cells (*I*) and HIV replication in sorted CCR6^+^/CCR6^−^ T cells are shown (*J*) as well as a statistical analysis of HIV-p24 levels in sorted CCR6^+^ T cells (*K*).

**Fig. 4.**
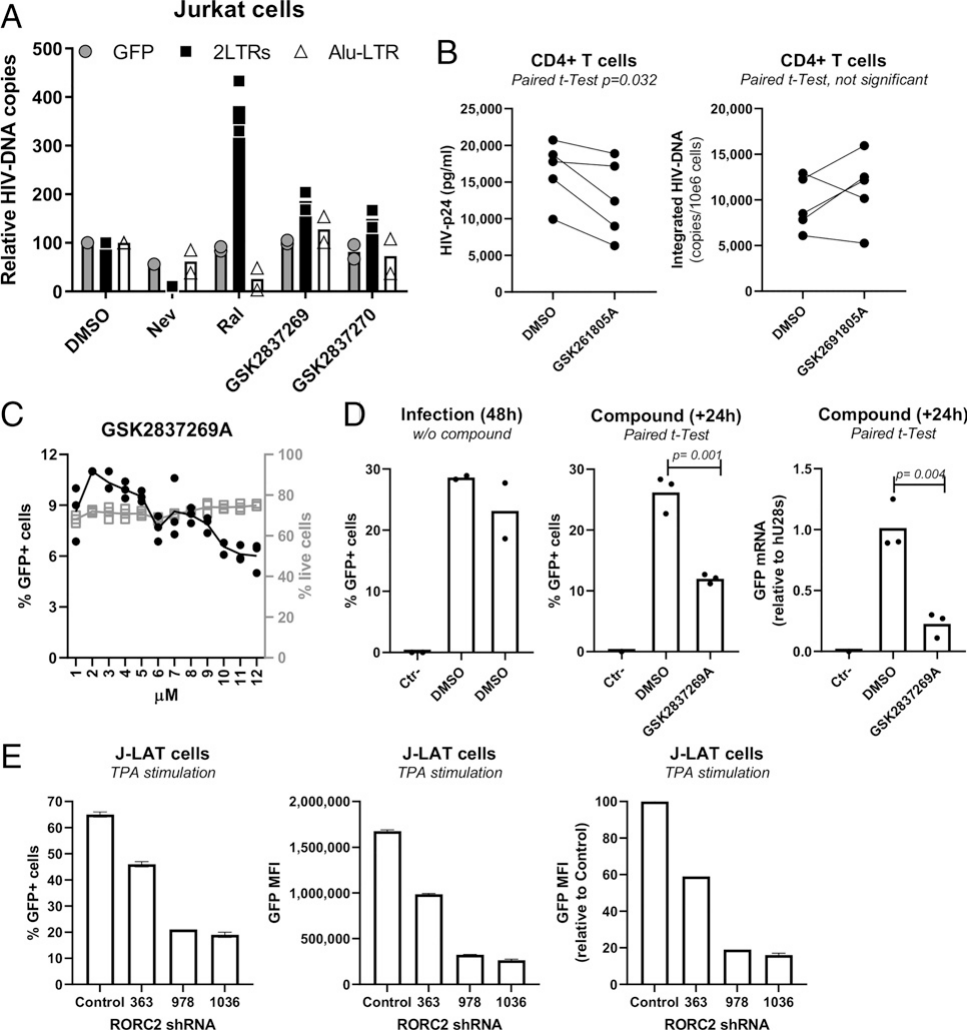
RORC2 promotes HIV-1 gene expression and binds to the HIV-1 LTR. (*A*) Jurkat cells were infected with VSV-G–pseudotyped single-cycle HIV-1 LAI_GFP_ in the presence of the reverse transcriptase inhibitor nevirapine (500 nM), the integrase inhibitor raltergravir (100 nM), or the indicated RORC inhibitors (5 μM). The amount of negative-strand viral DNA (GFP) and 2-LTR circular DNA was quantified 24 h postinfection by Taqman qPCR. Integrated viral DNA was quantified by Alu-LTR qPCR on DNA extracted 8 d after infection, *n* = 2. (*B*) Memory CD4^+^ T cells from *n* = 5 HIV− donors were stimulated with CD3/CD28 Abs in the presence or the absence of GSK261805A (5 μM) for 3 d and then infected with single round VSV-G–pseudotyped NL4.3HIV-1. The cells were cultured with IL-2 in the presence or the absence of GSK261805A (5 μM) for an additional 3 d. HIV-p24 levels in cell-culture supernatants measured by ELISA (*Left*) and integrated HIV-DNA levels (*Right*) quantified by nested real-time PCR at day 3 postinfection are shown. (*C*) Jurkat cells were infected with VSV-G–pseudotyped single-cycle HIV-1 LAI_GFP_, and 24 h postinfection, they were treated with the indicated concentrations of GSK2837269A. The cells were analyzed 24 h later by flow cytometry to determine the percentage of infected (GFP+) cells, *n* = 3. (*D*) Jurkat cells were infected with single-cycle HIV-1 LAI_GFP._ The cells were analyzed 40 h after infection by flow cytometry to determine the percentage of infected (GFP+) cells (*Left*). These cells were then treated with DMSO or GSK2837269A (5 μM) for 24 h before being reanalyzed by flow cytometry (*Middle*). RNA was extracted from the cells, and the amount of GFP mRNA relative to 28S ribosomal RNA was quantified by RT-qPCR. Average values are shown (*n* = 3) (*Left*). (*E*) J-Lat (clone A2) cells were transduced with the same RORC2-targeting shRNAs shown in Fig. 3*A*, selected with puromycin for 7 d, and stimulated with 12-O-Tetradecanoylphorbol-13-Acetate for 24 h before analysis by flow cytometry.

Next, we tested the activity of the RORC2 inhibitors on HIV-1 replication in primary CD4^+^ T cells. Memory CD4^+^ T cells were isolated from five HIV-uninfected healthy donors (HIV-), stimulated with CD3/CD28 antibodies (Abs), and infected with HIV-1_THRO,_ a transmitted/founder virus (8), in the presence of GSK2691805A or DMSO (34, 35) (Fig. 2*A***)**. We observed that GSK2691805A potently reduced HIV-1 replication (Fig. 2 *B*–*D*). The other RORC2 inhibitors GSK2837269A, GSK2793955A, and GSK2833332A also reduced HIV-1 replication at a concentration of 5 μM, although the differences did not reach statistical significance (Fig. 2 *E* and *F*). We observed some differences in the compounds’ potency to inhibit HIV replication between Jurkat and primary CD4^+^ T cells at the 5 μM concentration: GSK2837270A was active in Jurkat cells but not in primary CD4^+^, and conversely, GSK283332A was inactive in Jurkat cells but showed an antiviral activity in primary CD4^+^ T cells. GSK2837270A may be less potent in primary cells due to its different susceptibility to drug transporters and efflux pumps (36) or faster degradation in these cells. All compounds except for GSK2833332A reduced IL-17A but not IFN-γ production at 5 µM (*SI Appendix*, Fig. 2 *A* and *B*). This result suggested that GSK2833332A might inhibit HIV-1 infection in primary cells by a mechanism independent of RORC2. No cytotoxicity or changes in cell proliferation for any of the compounds was observed at the tested concentrations (*SI Appendix*, Fig. 3). Using the same experimental design, GSK2691805A also reduced replication of the primary isolate HIV_NL4.3BaL_, which is well adapted to grow in primary CD4^+^ T cells (*SI Appendix*, Fig. 4 *D* and *E*). Therefore, inhibition of RORC2 impairs the replication of different HIV strains in Jurkat and primary CD4^+^ T cell models.

**Fig. 5.**
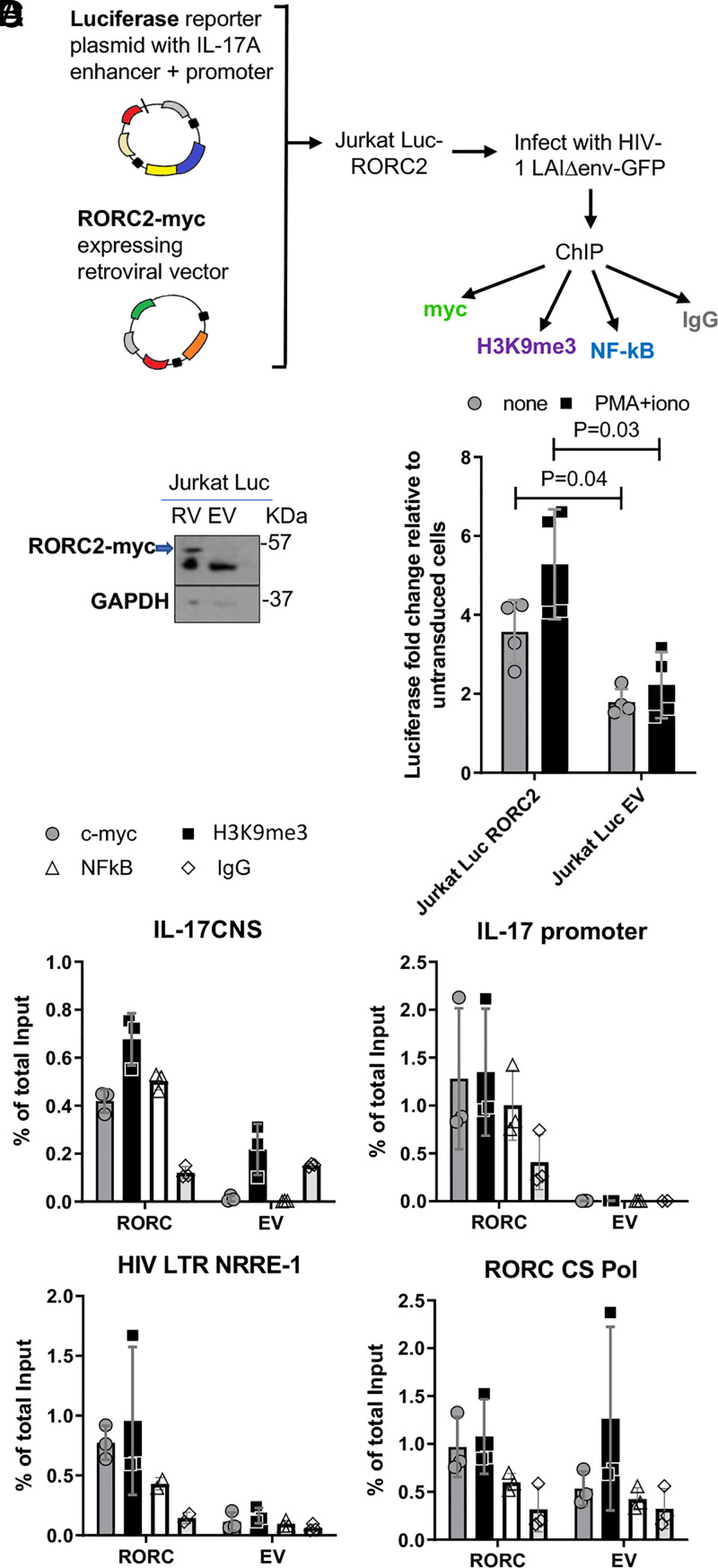
RORC2 binds to the HIV promoter. (*A*) A schematic diagram explaining the experimental steps for ChIP. Jurkat cells expressing luciferase driven by the IL-17A enhancer/promoter region were transduced with a retroviral vector expressing RORC2-myc (Luc-RORC2) or an EV control (Luc-EV). Luc-RORC2 and Luc-EV cells were infected in parallel with VSV-G–pseudotyped HIV-1 LAI ΔenvGFP (HIV-1_GFP_) and, 24 h later, processed for ChIP with the four indicated Abs. (*B*) Western blot with an anti-myc antibody to detect RORC-myc following ChIP (the lower band is IgG heavy chains); GAPDH in the total lysate samples was used as a loading control. (*C*) The fold change in Luciferase expression relative to untransduced Jurkat Luc cells with or without stimulation with PMA and ionomycin. The average ± SD, *n* = 3, *P* value based on two tailed Student’s *t* test is shown. (*D*) A real-time PCR signal following ChIP for the indicated DNA regions and antibody. (*Top Left*) IL-17A enhancer region. (*Top Right*) IL-17A promoter region. (*Bottom Left*) NRRE region in the HIV-1 LTR. (*Bottom Right*) HIV-1 pol region containing the putative RORC2 consensus element. The replicate values are shown as well as the average ± SD, *n* = 3.

The pool of memory CD4^+^ T cells includes a Th17-polarized fraction for which the expression of Th17 effector functions requires T cell receptor (TCR) triggering (10, 37). To determine if RORC2 expression in this Th17-polarized population promoted HIV-1 infection in vitro, memory CD4^+^ T cells from healthy donors were stimulated for 3 d and infected with HIV-1_THRO_ (*SI Appendix*, Fig. 5*A*). At 6 d postinfection, the cells were analyzed by flow cytometry to detect RORC2 and HIV-p24 expression. The percentage of HIV-p24^+^ cells was higher in RORC2^+^ than in RORC2^−^ cells in all five donors, indicating better HIV-1 infection rates in cells expressing RORC2 (*SI Appendix*, Fig. 5 *B* and *C*). In parallel, CD4^+^ T cells from these same donors were analyzed by flow cytometry to detect HIV-p24 expression in cells producing IL-17A and/or IFN-γ (IL-17A and IFN-γ are markers of Th17 cells and Th1 cells, respectively) upon simulation with phorbol 12-myristate 13-acetate PMA/Ionomycin (*SI Appendix*, Fig. 5 *D*–*F*). Notably, in these experimental settings, we confirmed that Th17- and Th17/Th1-polarized cells had a significantly higher proportion of HIV-p24^+^ cells than Th1 cells or unpolarized Th0 cells, which is consistent with their reported increased permissiveness to infection (10, 11).

To validate RORC2 as a host cofactor for HIV-1 infection, we generated three different hairpins targeting RORC2 messenger RNA (mRNA) and transduced them using lentiviral vectors into reporter Jurkat 1G5 cells, which express luciferase from the stably transfected HIV-1 LTR (38). We confirmed RORC2 knockdown (KD) by Western blot (Fig. 3*A*) and infected the cells with HIV-1_NL4.3_, which is well adapted to replicate in Jurkat cells. We found a reduced HIV-1 replication in KD cells relative to control cells, and this phenotype was proportional to the degree of RORC2 depletion, indicating specificity (Fig. 3*B*). To test if RORC2 was sufficient to promote HIV-1 infection, we expressed RORC2 complementary DNA (cDNA) in 293T cells, which do not normally express endogenous RORC2 (39) (Fig. 3*C*), and infected them with VSV-G–pseudotyped, single-cycle HIV-1 LAI_GFP_ in the presence of DMSO or the RORC2 inhibitor GSK2837269A, which had the best activity/toxicity profile in Jurkat cells (Fig. 1). Exogenous expression of RORC2 in 293T cells enhanced HIV-1 LAI_GFP_ infection above the already high basal levels, and this phenotype was abrogated by GSK2837269A (Fig. 3*D*). No inhibitory effect on infection was observed with GSK2837269A in control 293T cells at 12μM, consistent with the fact that they do not express the target. We also stably expressed a myc-tagged RORC2 cDNA in Jurkat cells using a retroviral vector construct and infected the cells with the HIV-1 LAI_GFP_ virus. We found that Jurkat cells expressing exogenous RORC2 sustained better infection relative to control cells (Fig. 3 *E* and *F*) in agreement with the 293T cells data. To confirm the specificity of the RORC2 drug action, HIV-1_THRO_–infected primary CD4^+^ T cells and ACH2 cells [a cell line carrying integrated HIV-DNA (40, 41)] were cultured in parallel in the presence or the absence of GSK261805A. As shown in *SI Appendix*, Fig. 1 *A* and *B*, ACH2 cells express insignificant levels of RORC2 and RORC1 mRNA compared to memory CD4^+^ T cells and should therefore be insensitive to the RORC2 inhibitors. As expected, GSK261805A inhibited HIV-1 infection in CD4^+^ T cells but not in ACH2 cells, confirming RORC2 as the target mediating the phenotype (Fig. 3 *G* and *H*).

**Fig. 6.**
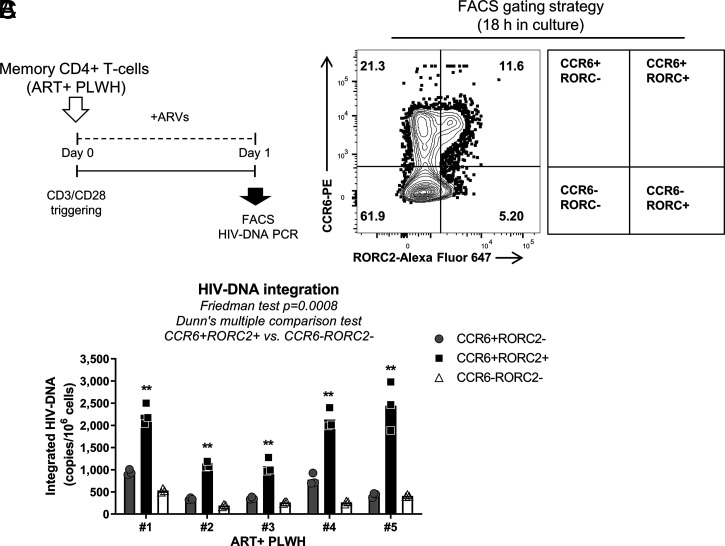
Memory CD4^+^ T cells expressing the Th17 markers CCR6 and RORC2 are enriched in integrated HIV-DNA in ART-treated PLWH. (*A*) A flowchart of the experimental approach; memory CD4^+^ T cells isolated from ART+ PLWH were stimulated with CD3/CD28 Abs for 18 h in the presence of antiretroviral drugs (AZT 180 nM, Efavirens 100 nM, and Raltegravir 200 nM) to prevent cell-to-cell HIV transmission in vitro. Highly pure CCR6^+^RORC2^−^, CCR6^+^RORC2^+^ and CCR6^−^RORC2^−^ cell subsets were sorted by FACS, and integrated HIV-DNA levels were quantified by nested real-time PCR. (*B*) The gating strategy used to sort the three cell populations mentioned in (*A*) and (*C*) the statistical analysis of integrated HIV-DNA in sorted CD4^+^ T cell subsets from *n* = 5 ART-treated PLWH. The individual replicates are shown with the bars representing median values.

We next sought to further validate RORC2 as a cofactor for HIV-1 infection by mRNA depletion in primary CD4^+^ T cells. Memory CD4^+^ T cells were CD3/CD28 stimulated for 2 d and then nucleofected with a small interfering RNA (siRNA) targeting RORC2 mRNA or a nontargeting control siRNA. A total of 1 d after nucleofection, the cells were infected with replication-competent HIV-1_NL4.3BaL_. At day 3 postinfection, we observed partial inhibition of the RORC2 mRNA expression in all the tested donors, which resulted in a lower IL-17A production from the treated cells relative to controls (*SI Appendix*, Fig. 6) and a small but significant reduction of HIV-1 infection as measured by the quantity of proviral DNA (Fig. 3*I*). Finally, because RORC2 is preferentially expressed by CCR6^+^ Th17 cells (42), RORC2 siRNA experiments were similarly performed on flow cytometry–sorted CCR6^+^ (Th17) and CCR6^−^ (non-Th17) CD4^+^ T cells. The highest levels of HIV replication were detected in CCR6^+^ T cells, which agrees with previous reports by our group and others (8, 18, 19, 21, 22). It is noteworthy that the depletion of RORC2 mRNA resulted in a significant reduction in proviral DNA and HIV-p24 levels in the CCR6^+^ subset with no effects observed in CCR6^−^ T cells, indicative of decreased HIV-1 replication upon RORC2 depletion (Fig. 3 *J* and *K*). Taken together, these pharmacological and genetic approaches demonstrated that RORC2 promotes HIV-1 replication.

### RORC2 Regulates HIV-1 Gene Expression.

To determine the step of the viral replication cycle that was impaired by the anti-RORC2 compounds, we infected Jurkat cells with single-cycle HIV-1 LAI_GFP_ at a multiplicity of infection (MOI) of 0.1 in the presence of GSK2837269A, GSK2837270A, nevirapine (a nonnucleoside inhibitor of reverse transcriptase), raltegravir (a strand transfer inhibitor of integration), or DMSO. Total DNA was extracted from the cells at 24 h postinfection, and Taqman qPCR was used to measure the amount of GFP DNA (as a surrogate marker of negative strand viral DNA) and 2LTRs circular DNA, a hallmark of nuclear entry (43). To measure the amount of integrated viral DNA, we performed Alu-LTR qPCR from DNA extracted 8 d postinfection (27). RORC2 inhibition did not significantly impair reverse transcription, nuclear entry, or integration in contrast to nevirapine, which reduced both viral DNA and 2LTRs, or raltegravir, which suppressed integration (Fig. 4*A*). We also infected primary CD4^+^ T cells with a single-round VSV-G–pseudotyped HIV (NL4.3 backbone, env-) in the presence of GSK2691805A and measured HIV-p24 levels by enzyme-linked immunosorbent assay (ELISA) and integrated HIV-DNA levels by nested real-time PCR 72 h postinfection. While HIV-p24 levels were reduced (Fig. 4 *B*, *Left*), the number of integrated HIV-DNA copies was not statistically different (Fig. 4 *B*, *Right*), indicating that RORC2 acts at a step postintegration such as gene expression.

To examine this aspect further, GSK2837269A was added on to Jurkat cells chronically infected with single-cycle HIV-1 LAI_GFP_, and the percentage of GFP+ cells was analyzed by flow cytometry 48 h later. A dose-dependent reduction of the percentage of GFP+ cells was observed (Fig. 4*C*). To examine viral gene expression directly, Jurkat cells were infected with single-cycle HIV-1 LAI_GFP_ in the absence of compounds and analyzed by flow cytometry 48 h later (Fig. 4 *D*, *Left*). Next, GSK2837269A was added onto these infected cells, which were reanalyzed by flow cytometry 24 h later (Fig. 4 *D*, *Middle*). In parallel, RNA was extracted from the GSK2837269A-treated cells and used for RT-qPCR to detect GFP mRNA, which is transcribed from the viral LTR. At 5 μM, GSK2837269A reduced GFP mRNA fivefold (Fig. 4 *D*, *Right*). The modest discrepancy between GFP mRNA and percentage of GFP+ cells is most likely explained by the longer half-life of the GFP protein relative to its mRNA. To confirm that RORC2 is important for HIV-1 gene expression, we transduced J-Lat cells (clone A2) (44), which harbor a latent HIV-1 provirus expressing GFP, with the same RORC2-targeting short hairpin RNAs (shRNAs) shown in Fig. 3*B*. Upon stimulation with 12-O-Tetradecanoylphorbol-13-Acetate, ≥60% J-Lat cells expressed GFP, while cells treated with the shRNAs showed lower GFP expression in terms of frequency and MOI, indicating that RORC2 silencing inhibited HIV-1 gene expression (Fig. 4 *E*–*G*).

**Fig. 7.**
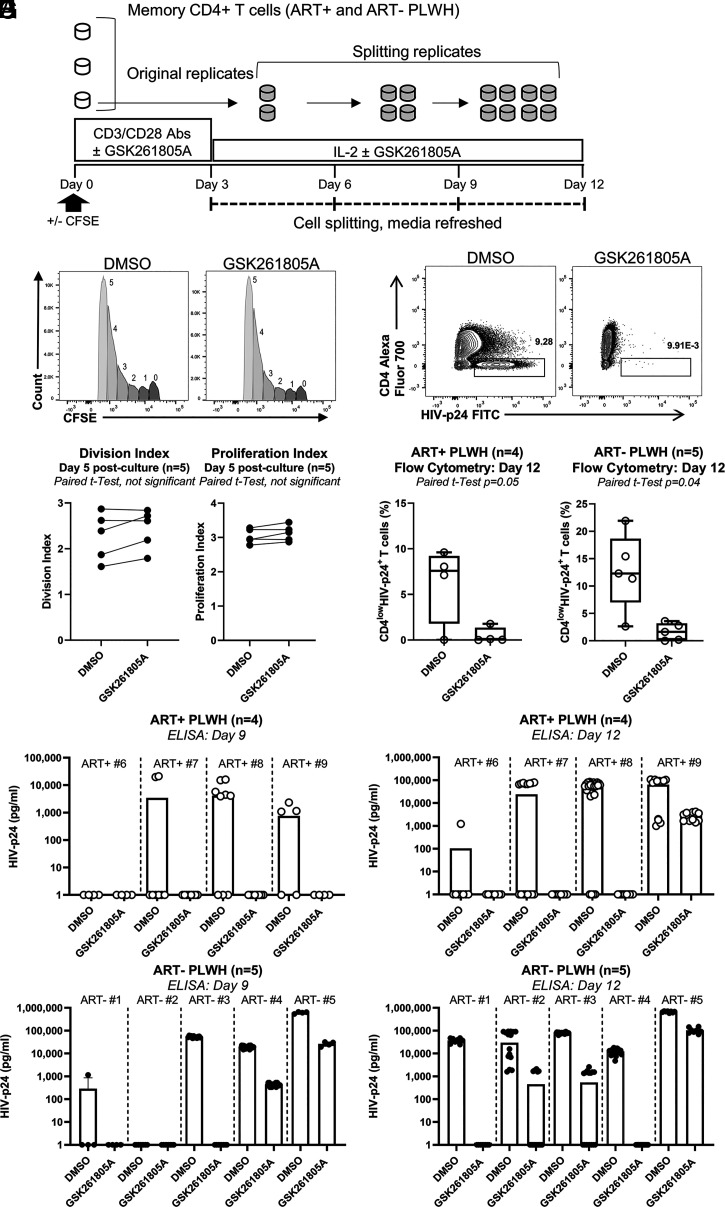
RORC antagonism inhibits HIV-1 outgrowth in memory CD4^+^ T cells of ART-treated and untreated PLWH. (*A*) An experimental flowchart of the viral outgrowth assay; memory CD4^+^ T cells isolated from PLWH receiving ART (ART+ PLWH) or not (ART− PLWH) were activated with CD3/CD28 Abs in the presence of DMSO or GSK2691805A (5 μM) at a cell concentration of 1 million cells/mL in triplicates for 3 d. Then, cells were washed and split at day 3 poststimulation and cultured in medium containing IL-2 (5 ng/mL) for up to 12 d (split every 3 d) in the presence of DMSO or GSK2691805A (5 μM). At day 12, HIV-infected cells were identified as CD4^low^HIV-p24^+^ by flow cytometry. HIV-p24 levels in cell-culture supernatants were measured by ELISA. (*B* and *C*) In parallel, using the same experimental setup, a VOA was performed with CFSE-loaded memory CD4^+^ T cells obtained from ART± PLWH. CFSE dilution was evaluated by flow cytometry at day 5 postactivation. A histogram of CFSE dilution showing cell divisions (division 0 to 5) of one representative individual (*B*) and a statistical analysis of the division and proliferation index obtained with cells of *n* = 5 ART ± PLWH are shown (*C*). (*D*) The frequency of CD4^low^HIV-p24^+^ cells in one representative individual. (*E* and *F*) The statistical analysis of results obtained with cells of *n* = 4 ART+ PLWH (*E*) and *n* = 5 ART− PLWH (*F*). (*G* and *H*) The effect of GSK2691805A on HIV-p24 levels in cell-culture supernatants of *n* = 4 ART+ PLWH (*G*) and *n* = 5 ART− PLWH (*H*) at days 9 and 12 post-CD3/CD28 activation. Paired Student’s *t* test *P* values are indicated on the graphs. Box and whiskers plots with individual values and maximum and minimum (*E* and *F*) and individual replicate values are shown with bars indicating median values (*G* and *H*).

RORC2 binds to specific DNA consensus elements, recruiting chromatin-activating cofactors via its LBD to regulate transcription (23, 45). We therefore hypothesized that RORC2 might bind to such DNA elements on the HIV-1 provirus. The HIV-1 LTR contains a well-characterized nuclear receptor responsive element (NRRE) that binds several retinoic acid receptors (46, 47). In addition, we have detected in silico a consensus RORC DNA-binding motif (23, 34) in the pol region of the HIV-1 genome (*SI Appendix*, Fig. 7). It has previously been reported that this region in Pol may have enhancer activity (48). To test the hypothesis, we performed chromatin immunoprecipitation followed by real-time PCR (ChIP-qPCR) for both the NRRE in the LTR and the RORC2 consensus sequence in Pol (CS Pol). There is no available ChIP-grade antibody against human RORC2. Hence, we performed the experiments in Jurkat cells stably transduced with a retroviral vector expressing C-terminally myc-tagged RORC2. However, Jurkat cells do not express IL-17A, raising the possibility that the IL-17A locus might be defective, thus making these cells unsuitable to detect RORC2 binding to the IL-17A regulatory elements by ChIP. To circumvent this problem, we transduced with the RORC2-myc expressing retroviral vector Jurkat cells stably transfected with a plasmid expressing luciferase driven by the IL-17A promoter and CNS-5 enhancer, which contains one RORC2 DNA consensus element each (Jurkat-Luc cells) (31, 49) (Fig. 5 *A* and *B*). Jurkat-Luc cells transduced with an empty retroviral vector (Jurkat Luc-EV) were generated to control for specificity. The resulting Jurkat Luc-RORC2 cells expressed luciferase at higher levels than Jurkat Luc-EV both at baseline and after stimulation, confirming their functionality (Fig. 5*C*). Jurkat Luc-RORC2 and Luc-EV cells were infected in parallel experiments with HIV-1 LAI_GFP_ at low MOI. The cells were processed for ChIP using an anti-myc antibody 24 h after infection. We also employed an antibody against H3K9me3, which is a histone marker of inactive but poised enhancer/promoters (50) and an antibody against NF-κB, a transcription factor known to bind both the HIV-1 LTR (51) and the IL-17 enhancer/promoter regions (52, 53). A specific ChIP signal for RORC2-myc was detected on the IL-17 enhancer and promoter regions of Jurkat Luc-RORC2 cells but not Jurkat Luc-EV cells (Fig. 5*D*). Notably, a specific ChIP-qPCR signal was observed on the HIV LTR NRRE element, whereas the signal for the CS Pol element was less convincing due to high background, which may be related to greater “stickiness” of the specific DNA sequence under study due to charge and/or secondary structure (Fig. 5*D*).

These results showed that RORC2 binds to the HIV-1 LTR with similar or even greater strength than the IL-17 promoter/enhancer regions. Notably, a specific ChIP-qPCR signal was also detected for H3K9me3 in Jurkat-Luc cells, particularly on the IL-17A enhancer/promoter region. This H3K9me3 signal was stronger in RORC2-expressing cells, suggesting that RORC2 might either associate with or increase the proportion of poised enhancers. It should be noted that Jurkat cells were not stimulated before ChIP, hence some critical RORC2 cofactors important for chromatin remodeling might be present at low levels (23, 31, 53), reducing the rate of enhancer conversion from poised into active. Furthermore, the RORC2-specific ChIP-qPCR signal is likely to be an underestimation due to competition with the endogenous RORC2, which is expressed in Jurkat cells (*SI Appendix*, Fig. 1) but cannot be precipitated by the anti-myc Ab. Consistent with previous reports, we did not detect NF-κB binding to the IL-17 enhancer/promoter in the absence of stimulation (52, 53); however, expression of RORC2 appeared to stimulate recruitment of NF-κB on both the IL-17 enhancer/promoter region and the HIV-1 LTR, which is in line with the observed cooperativity of RORC2 and some transcription factors (23, 31, 52). Taken together, these results support the possibility that RORC2 regulates viral gene expression by binding to the HIV-1 LTR.

### RORC2 Is Critical for HIV-1 Outgrowth from Patients’ Cells.

Since RORC2 promotes HIV-1 gene expression in acutely infected cells, we tested if its inhibition also prevented HIV-1 outgrowth from cells of PLWH. RORC2-mediated effector functions are not constitutive in Th17 cells but depend on TCR triggering (10, 37); thus, we initially evaluated if the activation of memory CD4^+^ T cells with CD3/CD28 Abs was capable of inducing the expression of RORC2 at the transcriptional and protein levels. Memory CD4^+^ T cells were isolated from HIV-uninfected individuals and stimulated for 5 or 24 h. The relative RORC2 mRNA and protein levels were assessed 5 or 24 h later, respectively. We observed that cell activation induced the expression of RORC2 but not RORC1 mRNA (*SI Appendix*, Fig. 8 *C* and *D*) . At the mRNA and protein level, RORC2 expression was up-regulated by TCR activation, and cells expressing RORC2 also expressed CCR6 (*SI Appendix*, Fig. 8 *C* and *D*) , which is a well-established marker of human Th17 cells (9) and HIV-reservoir enrichment (8, 10, 18, 19, 22). Next, to determine if cells expressing RORC2 harbor HIV-1, memory CD4^+^ T cells were isolated from ART+PLWH and stimulated in vitro with CD3/CD28 Abs for 24 h in the presence of antiretroviral therapies (ARVs) to prevent HIV cell-to-cell transmission (Fig. 6*A*). This stimulation is required for optimal expression of RORC2, which, similar to all lineage-specific cytokines, is not constitutively expressed in Th17-committed CCR6^+^ T cells (37). Cells were then sorted based on CCR6 and/or RORC2 expression (Fig. 6*B*) and analyzed by nested real-time PCR for HIV proviral DNA (Fig. 6*C*). In all five ART+ PLWH, proviral DNA was significantly more abundant in CCR6^+^RORC2^+^ cells compared to CCR6^−^RORC2^−^ cells or CCR6^+^RORC2^−^ cells (Fig. 6*C*). To explore whether CCR6^+^RORC2^+^ cells carry translational-competent HIV-DNA, we performed a modified HIV flow assay to quantify HIV-p24 expression in RORC2^+^ cells (54). To this end, memory CD4^+^ T cells isolated from ART- PLWH were stimulated for 72 h in the presence of ARVs, and RORC2 expression was measured in total T cells and productively infected CD4^low^HIV-p24^+^ T cells. As expected, we found that cell stimulation induced the expression of both RORC2 and HIV-p24 (*SI Appendix*, Fig. 9). Notably, CD4^low^HIV-p24^+^ T cells were enriched in RORC2 expression compared to total memory T cells (*SI Appendix*, Fig. 10 *A*–*C*). Additionally, CD4^low^HIV-p24^+^ T cells expressing RORC2 showed a higher HIV-p24 geometric mean fluorescence intensity compared to their RORC2^−^ counterparts (*SI Appendix*, Fig. 10*D*), which supports the notion that RORC2 stimulated HIV-1 gene expression.

Based on these observations, we sought to test if RORC2 inhibition prevented HIV-1 reactivation from latency and/or viral outgrowth ex vivo. To this end, we performed a simplified viral outgrowth assay (VOA) we have previously described (55) using memory CD4^+^ T cells from both ART+ and ART− PLWH. Cells were stimulated with CD3/CD28 Abs for 3 d and maintained in culture for another 9 d by splitting each well into two new wells every 3 d in the presence of DMSO or 5 μM GSK2691805A (Fig. 7*A*). Cells were analyzed to detect CD4^low^HIV-p24^+^ cells by flow cytometry at day 12 post-TCR triggering and the level of HIV-p24 in cell-culture supernatants was evaluated at days 9 and 12 postactivation by ELISA. In parallel, cell proliferation was evaluated by CFSE-based proliferation assay as described previously (56) on the cells from ART± PLWH. No difference on cell proliferation was observed between the DMSO and GSK2691805A conditions (Fig. 7 *B* and *C*). In the presence of GSK2691805A, there was a consistent reduction in the frequency of CD4^low^HIV-p24^+^ cells compared to DMSO on T cells from both ART+ and ART− PLWH (Fig. 7 *D*–*F*). Similarly, HIV-p24 levels in the culture supernatants were significantly reduced by GSK2691805A treatment (Fig. 7 *G* and *H*). These results are consistent with the notion that RORC2 is critical for HIV-1 reactivation/outgrowth in infected Th17 cells from PLWH.

## Discussion

In this study, we have identified the Th17 cell master transcription regulator RORC2 as a host cofactor for HIV-1 infection and showed that it regulates viral gene expression upon infection in vitro and possibly reactivation from latency as reflected by viral outgrowth ex vivo. These findings provide an explanation as to why HIV-1 replicates more efficiently in Th17 cells, which express higher levels of RORC2 relative to other CD4^+^ T cell types.

The ChIP-qPCR results indicate that RORC2 binds to the NRRE in the HIV-1 LTR. This DNA element is well conserved among different HIV-1 subtypes and was previously shown to be recognized by several nuclear hormone receptors, including retinoic acid receptors that positively regulate viral gene expression in CCR6^+^ Th17 cells (8, 11, 18). The NRRE in the HIV-1 LTR might be a critical element providing transcriptional plasticity to the virus, resulting in better adaptation to different cell types (57). It is noteworthy that estrogen inhibits HIV-1 reactivation from latency in Th17 cells (58). This effect is mediated by the binding of the nuclear hormone receptor estrogen receptor-1 (ESR-1) to the LTR, which may explain why women appear to have a lower inducible reservoir than men (58). Estrogen also inhibits Th17 cell differentiation and IL-17 secretion (59, 60), suggesting a broader interconnection between estrogen and RORC2. It would be interesting to test if RORC2 and ESR-1 compete for binding to the LTR and exert antagonistic effects on viral transcription.

We therefore propose that RORC2, rather than being essential for HIV-1 gene expression, acts more like a positive modulator of HIV-1 transcription, perhaps by helping establish a suitable chromatin environment. This may explain why HIV-1 replicates better in Th17 cells. Higher HIV-1 gene and protein expression in Th17 cells may also make these cells better targets for being killed by CD8^+^ T cells that, in part, explains long-term depletion of such cells from the GALT. In addition to a direct role in the modulation of HIV-1 gene expression, RORC2 may have other indirect effects that promote viral replication via the transcriptional regulation of other HIV-1 permissiveness factors (61).

Furthermore, our results demonstrate that the pharmacological inhibition of RORC2 potently suppressed HIV-1 outgrowth ex vivo in cells from ART-treated or untreated PLWH. This is consistent with the idea that RORC2 stimulates HIV-1 gene expression and may be important in regulating the dynamics of the viral reservoir (62). Fluctuations in RORC2 expression levels correlate with the activation status of Th17 cells, which is dependent, among other things, on priming via the TCR by specific antigens from pathogens such as *Candida albicans* and *Staphylococcus aureus* (9). Thus, specific stimuli from the microbiota may up-regulate RORC2 expression selectively in latently infected Th17 cells promoting HIV-1 reactivation from latency and viral rebound.

Pharmacological inhibition of RORC2 in vivo may help elucidate the contribution of Th17 cells on the latent viral reservoir. The GSK RORC2 inhibitors have been employed in animal studies for non-HIV indications, and GSK2691805A has been used to study the effect of RORC2 inhibition on Th17 and ILC-3 cells in mice (63). It will therefore be possible to conduct experiments in preclinical animal models of HIV-1 infection to determine if RORC2 inhibition delays or prevents viral rebound after ART interruption and/or whether RORC2 inhibition reduces inflammation and immune activation. A limitation of this approach is that Th17 cell differentiation and their effector functions might also be affected by administration of the RORC2 inhibitors. Nevertheless, the effects of RORC2 drugs may be reversible as indicated by the capacity of cells to produce IL-17 again after drug withdrawal in vitro. Optimal dosing regimens and regular monitoring of Th17 cell populations in various tissues may allow the safe testing of RORC2 inhibitors in preclinical and clinical interventions. Other potential limitations are linked to the fact that other cells also express RORC2, including ILC-3 and thymocytes (64, 65), although ILC-3 seems tolerant to prolonged inhibition of RORC2 (64). In consequence, these RORC2^+^ cells will need to be carefully monitored too. Despite these potential obstacles, which may be mitigated, targeting RORC2 in vivo may be a conceptually new approach to attenuate the loss of Th17 cells, which occurs during acute HIV-1 infection (10, 11)

## Materials and Methods

### Ethics Statement.

The collection of leukapheresis from HIV-uninfected individuals, ART− PLWH, and ART+ PLWH was conducted in compliance with the principles included in the Declaration of Helsinki. This study received approval from the Institutional Review Board (IRB) of the McGill University Health Centre and the Centre de recherche du Centre Hospitalier de l’Université de Montréal (CHUM). Written informed consents were obtained from all study participants.

### Human Subjects.

The human biological samples were sourced ethically, and their research use was in accord with the terms of the informed consents under an institutional review board (IRB)/ethics committee (EC)- approved protocol. HIV-uninfected individuals [HIV−; n = 18; 17 males and 1 female; median age of 57.5 y (range: 25 to 70), median CD4 counts 752 cells/μL (range: 511 to 1,115)] as well as virally suppressed ART-treated PLWH [ART+ PLWH; *n* = 9; 9 males and 0 female; median age of 44 y (range: 30 to 57), median CD4 counts 514 cells/μL (range: 318 to 598)] and untreated PLWH [ART− PLWH; *n* = 6; 6 males and 0 female; median age of 41 y (range: 24 to 50), median CD4 counts 459 cells/μL (range: 221 to 1,068)] (*SI Appendix*, Table 2) were recruited at the Montreal Chest Institute, McGill University Health Centre and CHUM. Peripheral blood mononuclear cells (PBMC) (10^9^ to 10^10^ cells) were collected by leukapheresis and frozen until use as previously described (8, 18, 20). Plasma viral load in ART-treated PLWH was measured using the Amplicor HIV-1 monitor ultrasensitive method (Roche).

### HIV Infection In Vitro of Primary Memory CD4^+^ T Cell.

The molecular clones of CCR5-tropic HIV-1 strain used in this study were the following: transmitted Founder (T/F) THRO and NL4.3BAL HIV-1. The T/F THRO HIV plasmid was obtained through the NIH AIDS Reagent Program, Division of AIDS, National Institute of Allergy and Infectious Diseases (NIAID), NIH: pTHRO.c/2626 (category no. 11745) from John Kappes and Christina Ochsenbauer. The NL4.3BaL HIV plasmid was provided by Michel Tremblay, Univerité Laval, Québec, Canada, originating from Roger J. Pomerantz, Thomas Jefferson University, Philadelphia, PA. The HIV-1 plasmid was amplified by MiniPrep and MaxiPrep and viral stocks were produced and titrated as we described it previously (8, 20). Memory CD4^+^ T cells were isolated from the PBMCs of HIV-uninfected individuals by negative selection using magnetic beads (Miltenyi Biotec) as we previously described (8, 20). Then, memory CD4^+^ T cells (1 × 10^6^ cells/mL per 48-well plate) were stimulated with immobilized CD3 and soluble CD28 Abs (1 µg/mL) for 3 d prior to infection. T cells were cultured with Roswell Park Memorial Institute (RPMI) 1640 media (Gibco) containing 10% fetal bovine serum (FBS) and 1% penicillin/streptomycin (P/S). Memory CD4^+^ T cells were infected with HIV-1 (20 to 50 ng HIV-p24/10^6^ cells) and then cultured in the presence of IL-2 (5 ng/mL; R&D Systems) for up to 9 d, with 50% of the media being refreshed every 3 d. Viral replication was measured by flow cytometry analysis upon HIV-p24 and CD4 staining (HIV-infected cells were identified as HIV-p24± and CD4^low^) as well as by HIV-p24 ELISA in cell-culture supernatant as previously described (18, 27).

### Cell Lines and Viruses.

293T cells (European Collection of Authenticated Cell Culture [ECACC], Public Health England) were grown in Dulbecco’s modified Eagle’s medium (Gibco Labs) supplemented with 10% fetal calf serum (FCS) (Helena Bioscience) and 2 mM glutamine (Gibco Labs) at 37 °C in 5% CO_2_. Jurkat E6.1 (ECACC) and Jurkat indicator line 1G5 containing the firefly luciferase gene driven by the HIV LTR (AIDS Research and Reference Reagent Program, Division of AIDS, NIAID, NIH from Estuardo Aguilar-Cordova and John Belmont) were grown in RPMI medium (Gibco Labs) supplemented with 10% FCS at 37 °C in 10% CO_2_. Jurkat cell line IL-17CNS luciferase clone 539 (GSK BIOCAT128253) (here called Jurkat Luc) was generated by transfection of Jurkat E6.1 cells with the plasmid pGL4-huIL-17 3-K CNS promoter containing the luciferase gene driven by the 1-Kb IL-17 CNS-5 enhancer fused to the 2-Kb promoter regions and were grown in RPMI medium with 10% FCS and 200 μg/mL hygromycin at 37 °C in 10% CO_2_. Viral stocks were prepared by Fugene transfection of 293T cells as described previously (43) using pHIV LAIΔenv (gift of Michael Emerman, Fred Hutchinson Cancer Research Centre, Seattle, WA) and pMD.G expressing VSV-G or using HIV isolate NL4.3 (Centre for AIDS Reagents, Health Protection Agency). Supernatant containing viral particles was collected 48 and 72 h posttransfection as described (43, 66). For infections, 13 mL 1G5 indicator cells (∼0.9 × 10^6^/mL) were mixed with 2 mL NL4-3 supernatant. The mix was dispensed robotically, 45 μL/well onto 384 plates preloaded with drug dilutions. Samples were analyzed 48 h postinfection using the BrightGlo assay according to the manufacturer’s instructions in a Pherastar plate reader. To generate Jurkat and 293T cells stably expressing human RORC2, the myc-DDK–tagged RORC2 cDNA was PCR amplified from plasmid RC212239 (Origene) and cloned into the murine leukemia virus-based retroviral vector pMIG Blasti (gift of Jeremy Luban, University of Massachusetts, Amherst, MA). The virus was produced in 293T cells as described and used to transduce Jurkat or 293T cells, which were selected in the presence of 5 μM blasticidin for 10 d. ACH2 cells were obtained through the NIH HIV Reagent Program, Division of AIDS, NIAID, NIH ACH-2 cells, ARP-349, contributed by Thomas Folks. ACH2 cells were kept in culture with RPMI, 10% FBS, and 1% P/S.

### Flow Cytometry Staining and Fluorescence-Activated Cell Sorting.

The following fluorochrome-conjugated Abs were used for flow cytometry analysis: HIV-p24 FITC (KC57) (Beckman Coulter), HIV-p24 PE (KC57) (Beckman Coulter), CD3 Pacific blue (UCHT1), CD4 PerCP/Cy5.5 (RPA-T4) (BioLegend), CD4 Alexa Fluor 700 (RPA-T4), CCR6 PE (11A9), CD45RA Alexa eFluor 780 (HI100), RORC2 Alexa Fluor 647 (Q31-378), *K*_i_-67 BUV395 (B56), IL-17A PE (eBio64DEC17), and IFN-γ Alexa Fluor 700 (B27). The Live/Dead Fixable Aqua Dead Cell Stain Kit (Vivid, Life Technologies) was used to exclude dead cells. Intracellular staining was performed using the BD Cytofix/Cytoperm Kit (BD Biosciences), and intranuclear staining was performed using the eBioscience Foxp3/Transcription Factor Staining Buffer Set. Cells were analyzed with the BD-LSRII cytometer, BD LSRFortessa and BD-Diva (BD Biosciences), and FlowJo version 10 (Tree Star, Inc.). The positivity gates were placed using fluorescence minus one strategy (8, 20). For fluorescence-activated cell sorting (FACS), memory CD4 T cells from the PBMCs of ART+ PLWH were isolated by negative selection using magnetic beads. CCR6^+^RORC2^+^, CCR6^+^RORC2^−^, and CCR6^−^RORC^−^ T cells were sorted by FACS (BDAria II; BD Biosciences) using the Abs CD3 Pacific blue (UCHT1), CCR6 PE (11A9), CD45RA Alexa eFluor 780 (HI100), and RORC2 Alexa Fluor 647 (Q31-378).

### Compounds.

GSK2793955A, GSK2805956A, GSK2833332A, GSK2837269A, and GSK2837270A were provided by GlaxoSmithKline at 100 mM stock in DMSO or freeze dried and reconstituted in DMSO. GSK2691805A was synthesized in house according to ref. 35 (*SI Appendix*, *Materials and Methods*).

### ChIP-qPCR.

ChIP assays were performed as described previously (66) with some modifications. Briefly, 5 × 10^7^ Jurkat cells expressing myc-tagged RORC2 or the pMI-blasti “empty” vector (EV) were infected with the VSV-G–pseudotyped LAIΔenv-GFP at an MOI of 0.3. After 24 h, the cells were collected in 50-mL tubes and chemically cross-linked by the addition of 1/10 volume of fresh 11% formaldehyde solution added directly to cell culture media and incubated for 20 min at room temperature with gentle rotation followed by the addition of 1/20 volume of cold 2.5 M glycine and incubated at 4 °C for 5 min. Cells were then collected by centrifugation at 4 °C, and the pellet was rinsed twice with phosphate buffered saline and flash frozen in liquid N_2_. Cells were resuspended and lysed in 1 mL lysis buffer one (50 mM Hepes-KOH, pH 7.5, 140 mM NaCl, 1 mM ethylenediaminetetraacetic acid (EDTA), 10% glycerol, 0.5% IGEPAL, and 0.25% Triton X-100) for 10 min at 4 °C with slow rotation. Nuclei were pelleted by centrifugation and gently resuspended in 1 mL Nuclei Wash buffer (200 mM NaCl, 1 mM EDTA) for 10 min at 4 °C with gentle rotation. Nuclei were pelleted by centrifugation and 1 mL lysis buffer three (LB3) (10 mM Tris, pH 8, 100 mM NaCl, 1 mM EDTA, 0.5 mM EGTA, 0.1% sodium deoxycholate, and 0.5% *N*-laurylsarcosine) was added without disturbing the pellet followed by incubation for 10 min at 4 °C with gentle rotation. This step was repeated once, and then the nuclei were pelleted by centrifugation, resuspended in 300 μL LB3, and kept on ice for 10 min. Samples were sonicated using a Diagenode Bioruptor device (30-s pulse/90-s pause × 10 cycles). Samples were centrifuged at 13,000 rpm for 6 min at 4 °C, and supernatant was collected in precooled 1.5-mL tubes. The resulting whole-cell extract (WCE) was incubated overnight at 4 °C with 100 μL protein-G magnetic Dynabeads preincubated with 10 μg appropriate antibody for 3 h on a rotating platform (9 rpm) in a cold room. The following antibodies were used: rabbit polyclonal anti-H3K9me3 (abcam, ab8898), normal rabbit IgG (Merk Millipore, 12–370), mouse mAb anti-c-Myc (Thermo Fisher, 9E10), and rabbit polyclonal anti-NF-κB p65 acetyl K310 (Abcam ab19870). The next day, the beads were washed (5 min with slow rotation) two times with a low salt buffer (10 mM Tris HCl pH 8, 150 mM NaCl, 1 mM EDTA, 1% Triton X-100, 0.1% sodium dodecyl sulfate [SDS], and phenylmethylsulfonyl fluoride [PMSF]), then once with a high salt buffer (10 mM Tris HCl pH 8, 500 mM NaCl, 1 mM EDTA, 1% Triton X-100, 0.1% SDS, and PMSF), then once with an LiCl buffer (10 mM Tris HCl pH 8, 1 mM EDTA, 0.5 mM EGTA, 250 mM LiCl, 1% IGEPAL, 1% NaDOC, and PMSF), then once with a Tris-EDTA (TE) buffer, and finally with elution buffer (TE + 1% SDS). Bound complexes were eluted from the beads by heating at 65 °C with occasional vortexing, and cross-linking in the immunoprecipitation and WCE samples was reversed by incubating at 65 °C for 6 to 7 h. Immunoprecipitation and WCE DNA were then purified by treatment with ribonuclease A and proteinase K and extracted with phenol/chloroform/isoamyl alcohol extractions. ChIP products were quantified by real-time qPCR. Primer sequences were as follows: IL-17 enhancer forward: 5′-TGATAGCCCAACCACAATGTG – 3′ (IL-17 gene nucleotide [nt] 1051 to 1072). IL-17 enhancer reverse: 5′- ACCTATACGTTAGCAGGCACA – 3′ (IL-17 gene nt 1220 to 1241). IL-17 promoter forward: 5′- TCTGCCCTTCCCATTTTCCT-3′ (IL-17 gene nt 2886 to 2906)

IL-17 promoter reverse: 5′- ATGGATGAGTTTGTGCCTGC-3′ nt 3064 to 3084. NRRE-1 in the HIV-1 LTR forward 5′-TCTACCACACACAAGGCTACT-3′, reverse 5′-ACAAGCTGGTGTTCTCTCCT-3′; RORC2 consensus sequence (CS) in HIV-1 Pol forward: 5′-GGGAAAGCTAGGGGATGGTT-3′ (HIV-1 nt 5137 to 5157), RORC2 CS reverse: 5′-TCAGGGTCTACTTGTGTGCT -3′ (HIV-1 nt 5322 to 5342). Real-time PCR was carried out in an Eppendorf Realplex in a final volume of 20 µL containing 1× SYBR green master mix (Applied Biosystems), 0.4 µM each primer, and 2 µL DNA (prediluted 1:10). The cycle parameters were the following: 95 °C for 2 min for 1 cycle followed by 95 °C for 1 min, 55 °C for 55 s, and 65 °C for 1 min 30 s for 45 cycles. The ChIP signal was calculated using the percent input method.

### VOA.

A simplified VOA was performed as we previously described (55). Briefly, memory CD4^+^ T cells were cultured at 1 × 10^6^ cells/well in 1 mL media (RPMI, 10% FBS, 1% P/S) in a 48-well plate (Costar) coated with CD3 Abs (1 μg/mL; BD Biosciences, Clone UCHT1) and in the presence of soluble CD28 Abs (1 μg/mL; BD Biosciences, Clone CD28.2). At day 3 poststimulation, cells from each original replicate were individually washed with media, split into two new CD3 Abs-uncoated 48-well plates, and cultured in media containing IL-2 (5 ng/mL; R&D Systems) in the presence or in the absence of GGSK2691805A (5 μM). The cells from each well were further split into two new wells (without washing) at day 6 and 9 poststimulation, with 50% of the media being refreshed with IL-2 with/without GSK2691805A. The cells were kept in culture for a total of 12 d.

### Statistical Analysis.

Statistical analyses were performed with GraphPad Prism 7 software (GraphPad Software, Inc.). One-way ANOVA and Friedman along with Dunnett’s and Dunn’s multiple comparisons test, respectively, evaluated the statistical differences. The use of nonparametric tests is justified by the fact that data sets did not pass the normal distribution test of Kolmogorov–Smirnov. *P* values ≤ 0.05 were arbitrarily considered statistically significant.

## Supplementary Material

Supplementary File

## Data Availability

Source numerical data files and images for all figures have been deposited within the University College London Data Repository (https://doi.org/10.5522/04/14892822.v1). GSK compounds GSK2837270A, GSK2793955A, GSK283726, GSK2833332A, and GSK2805956A are available upon reasonable request. GSK2691805 is commercially available at Calbiochem (https://www.sigmaaldrich.com/GB/en/product/mm/531369?gclid=EAIaIQobChMImtfX-pKT9AIVWe3tCh1EuATLEAAYAyAAEgLgH_D_BwE).
