# Supplementary material for: Th17 cell master transcription factor RORC2 regulates HIV-1 gene expression and viral outgrowth
Source: Proc Natl Acad Sci U S A. 2021 Nov 24;118(48):e2105927118. doi: 10.1073/pnas.2105927118 (PMC8640723; doi:10.1073/pnas.2105927118)
Supplement: Supplementary File [file pnas.2105927118.sapp.pdf]

Supporting Information Table 1: RORC2 compounds inhibit HTRF-ligand binding and Jurkat IL-17 luciferase reporter expression.

	HTRF-Ligand Binding		Jurkat IL-17 Luciferase	
**Compound**	**IC50 Mean µM (+/- SD), n**	**Vmax Mean (+/- SD), n**	**IC50 Mean µM (+/- SD), n**	**Vmax Mean (+/- SD), n**
GSK2691805A	0.039 (0.001), 3	38 (10), 3	0.040 (0.002), 3	110 (0), 3
GSK2837270A	0.016 (<0.001), 2	94 (3), 2	0.501 (0.025), 2	110 (0), 2
GSK2793955A	0.025 (0), 1	86 (0), 1	0.794 (0.010), 2	110 (0), 2
GSK2833332A	>10 (0), 1	-	-	-
GSK2805956A	>10 (0), 1	-	-	-
GSK2837269A	0.016 (<0.001), 2	94 (4), 2	0.079 (0.001), 4	110 (1), 4

Supporting information Table 2: Clinical parameters of HIV-infected untreated and ART-treated study participants.

ID	Sex	Age″	CD4 count#	CD4:CD8 Ratios	Viral load&	Time since infection*	ART	Time on ART*
ART+ #1	M	45	318	0,7	<40	150	Delaviridine Kivexa	50
ART+ #2	M	44	459	0,8	<40	189	Truvada Raltegravir	-
ART+ #3	M	46	581	0,5	<40	99	-	94
ART+ #4	M	32	523	1,0	<40	52	Truvada Reyataz	48
ART+ #5	M	57	514	0,9	<40	16	Tivicay Truvada	11
ART+ #6	M	44	398	0,5	<40	154	Complera	25
ART+ #7	M	36	542	0,7	<40	13	Stribild	12
ART+ #8	M	49	458	0,5	<40	227	Truvada Viramune	201
ART+ #9	M	30	598	1,0	<40	80	Stribild	77
ART- #1	M	24	316	0,5	9,496	55	None	N.A
ART- #2	M	47	529	1,2	3,189	110	None	N.A
ART- #3	M	42	221	0,5	41,774	89	None	N.A
ART- #4	M	50	389	0,3	97,552	150	None	N.A
ART- #5	M	40	1,068	1,2	22,812	2	None	N.A
ART- #6	M	24	897	1,3	18,621	-	None	N.A

M, male; F, female; ART-, ART-untreated PLWH; ART+, ART-treated PLWH; ″, years; #, cells/µl; &, HIV-RNA copies/ml plasma; *, months on ART; -, information not available; N.A, not applicable

Supporting information Figures

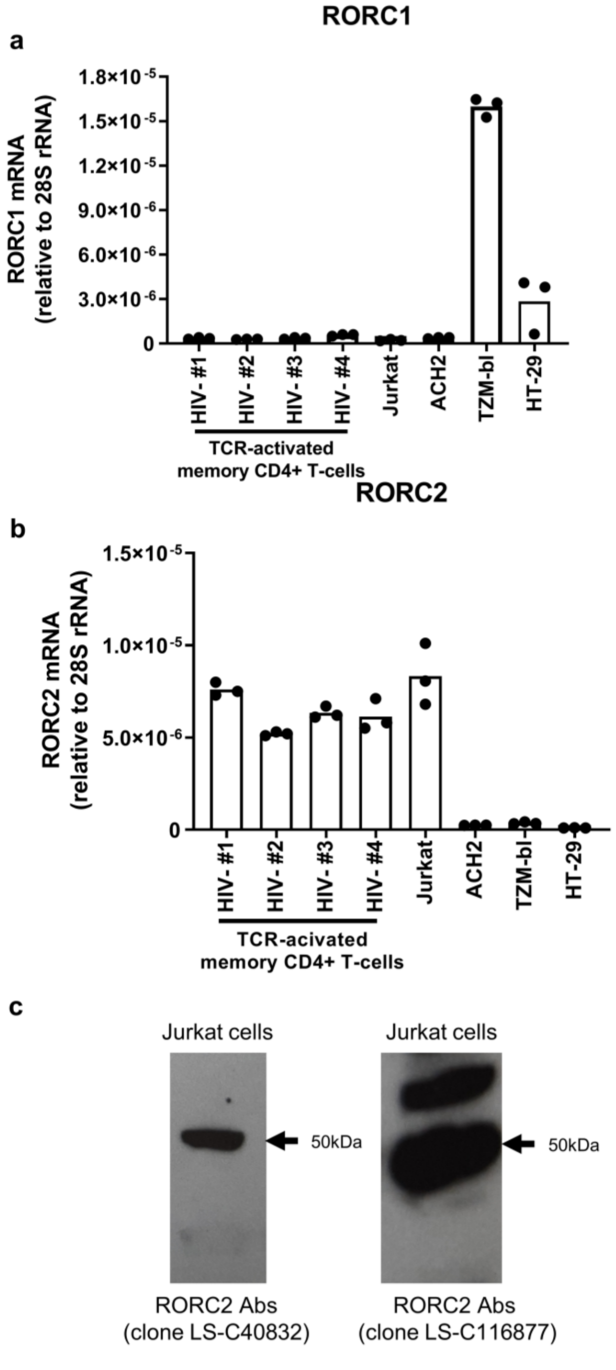

Supporting information Figure 1. **RORC2, but not RORC1, is expressed in primary CD4+ T cellsand Jurkat cells**. **(a-b)** The relative gene expression of RORC1 mRNA **(a)** and RORC2 mRNA **(b)** wasevaluated by real-time RT-qPCR in CD3/CD28-activated memory $CD4^+$ T cells isolated from n=4 HIV- uninfected individuals as well as in the Jurkat, ACH2, TZM-bl and HT-29 cell lines. **(c)** Western blot of Jurkat cell lysates to detect RORC2 expression. The antibody used is indicated below each panel.

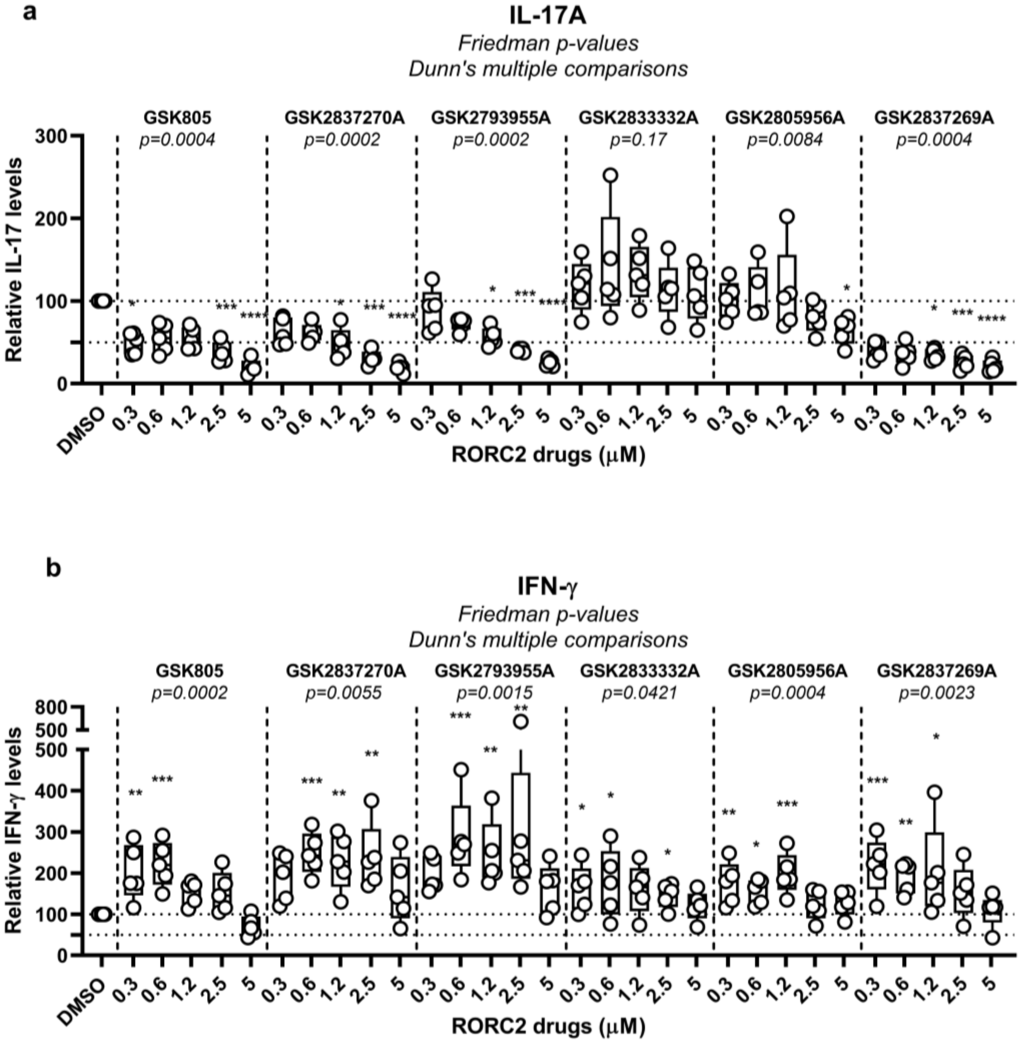

Supporting information Figure 2. RORC2 inhibitors reduce IL-17A, but not IFN-γ production. Memory CD4+ T cells were isolated and stimulated with CD3/CD28 Abs in the presence or absence ofthe indicated RORC2 inhibitors, as described in Figure 2. Levels of IL-17A and IFN-γ were measured by ELISA at day 3 post-stimulation. Statistical analysis of IL-17A **(a)** and IFN-γ **(b)** levels in cell culture supernatants of experiments performed with cells from n=5 HIV-uninfected individuals. Friedman p- values, with Dunn's multiple comparison significance, are indicated on the graphs (*, $p<0.05$; **, $p<0.01$;
***, $p<0.001$; ****, $p<0.0001$).

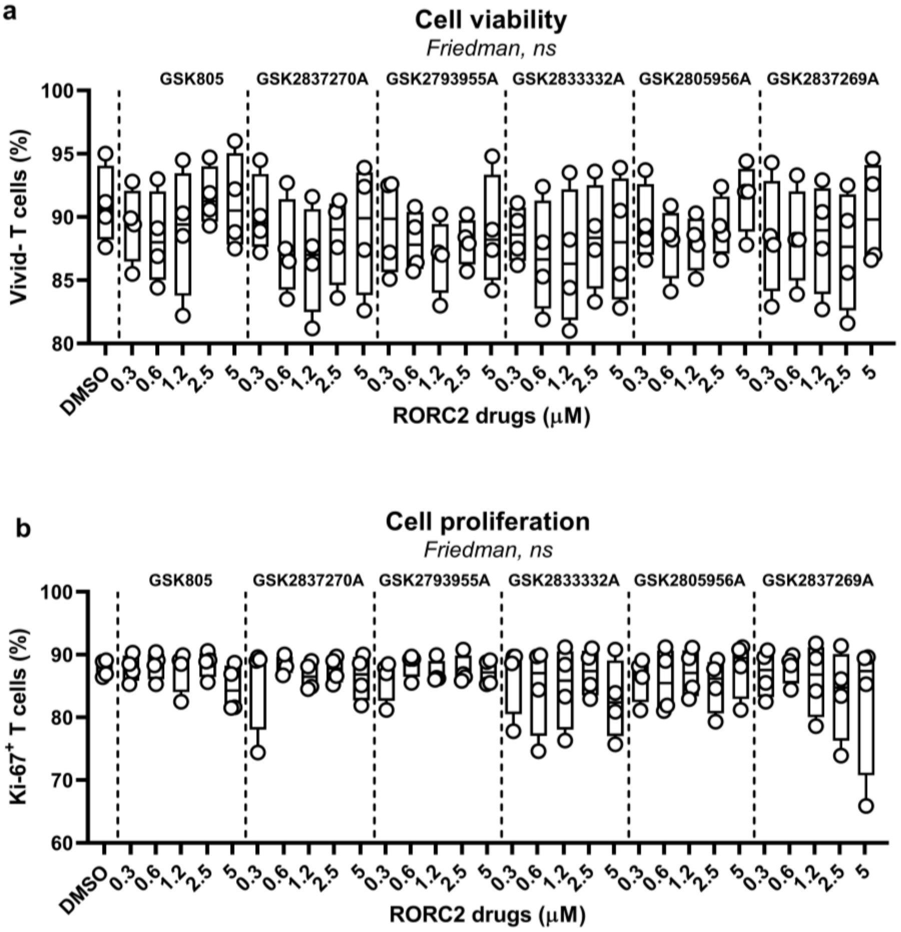

Supporting information Figure 3. No effect of RORC2 inhibitors on cell viability and proliferation. A fraction of memory $CD4^+$ T cells from experiments depicted in **Figure 2** were harvested 3 days after CD3/CD28 stimulation and stained with Live/dead Fixable Aqua dead stain kit and fluorochrome-conjugated Ki-67 Abs for flow cytometry analysis. Shown are the statistical analysis of the frequency of Live (Vivid-) **(a)** and Ki-67+ cells **(b)** in experiments performed with cells from n=5 HIV-uninfected individuals.

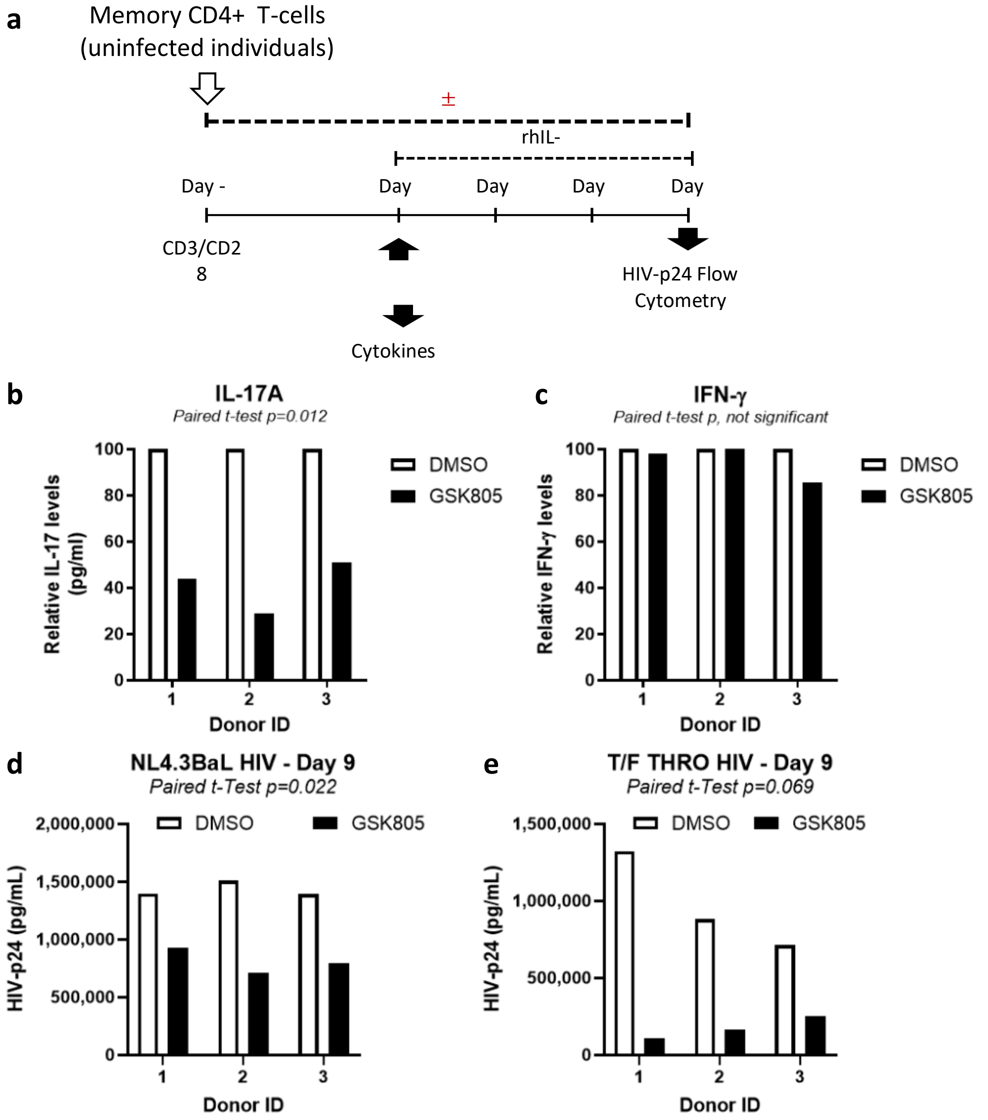

Supporting information Figure 4. The RORC inhibitor GSK261805 decreases $HIV_{NL4.3BaL}$ replication in memory $CD4^+$ T cells. Shown is the effect of GSK261805 on IL-17A **(b)** and IFN-γ **(c)** production in memory $CD4^+$ T cells of n=3 HIV- participants at D3 post-TCR riggering (prior to infection), as well as replication of $HIV_{NL4.3BaL}$ **(d)** and HIV_{THRO} **(e)** strains at 9 days post-infection

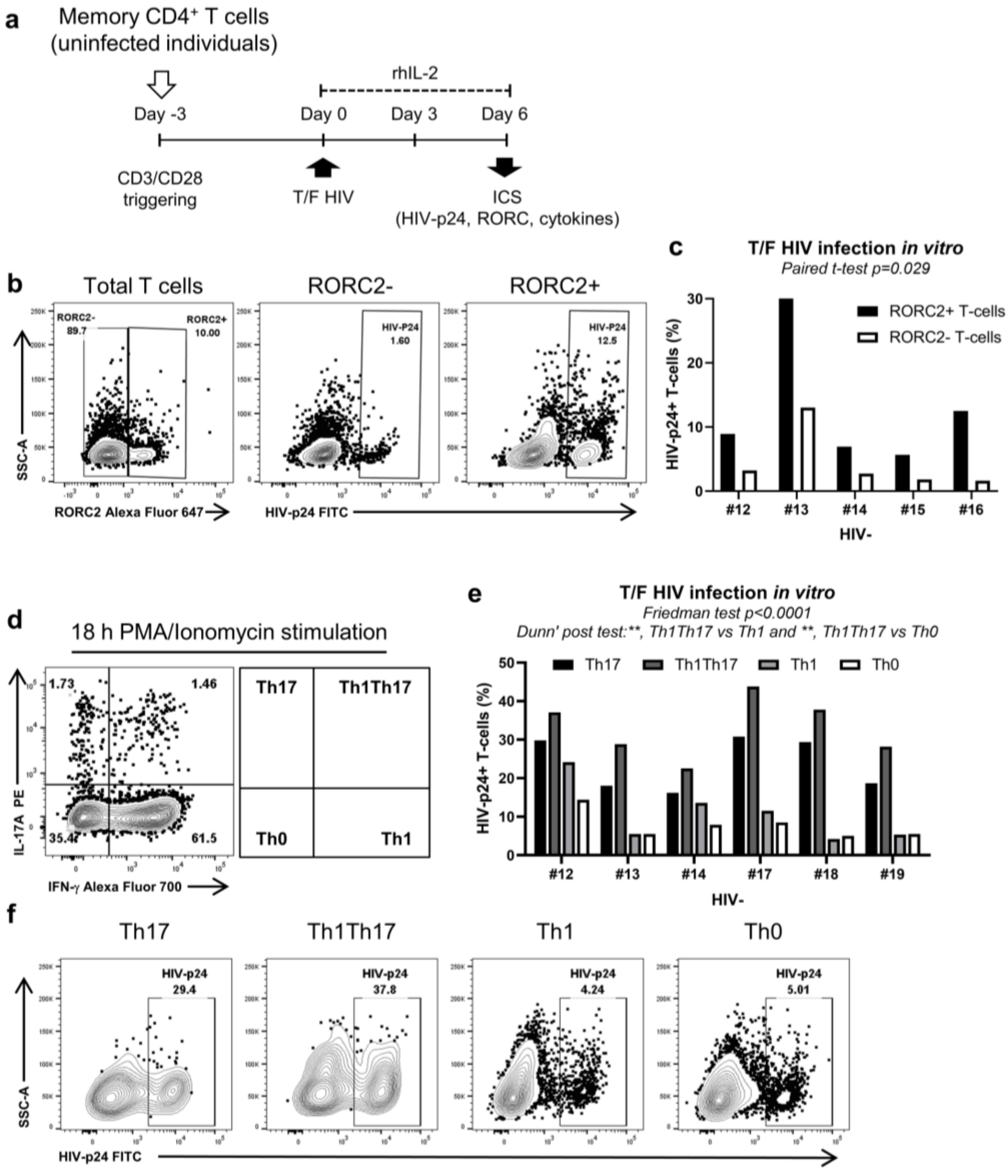

Supporting information Figure 5. **Preferential HIV-1 replication in Th17-polarized RORC2^{+} cells *in vitro*.** Experimental flow chart; memory CD4^{+} T cells isolated from n=5 HIV-uninfected individualswere stimulated with CD3/CD28 Abs for 3 days and exposed to HIVTHRO. After infection, cells were cultured in the presence of IL-2 for 6 days. The intracellular expression of HIV-p24, RORC2 and cytokines (IL-17A and IFN-γ) was analysed by flow cytometry **(a)**. Shown are contour plots of RORC2 expression **(b, left panel)**, HIV-p24 expression in RORC2^{-} and RORC2^{+} cells of one representative individual **(b, middle/right panels respectively)** and statistical analysis of results obtained with cells form n=5 individuals **(c)**. Gating strategy used to identify Th17 (IL-17A+IFN-γ-), Th1Th17 (IL-17A+IFN-γ+), Th1 (IL-17A-IFN-γ+), Th0 (IL-17A-IFN-γ-) **(d).** Shown are the contour plots of HIV-p24 expression in Th subsets of onerepresentative individual **(e)** and statistical analysis of experiments performed with cells from n=6 individuals **(f)**.

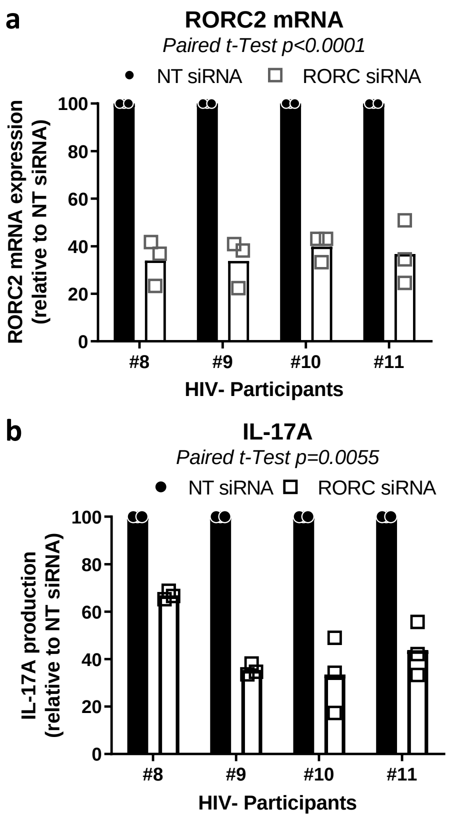

Supporting information Figure 6. Effect of RORC2 RNA interference on RORC2 and IL-17A expression. Memory $CD4^+$ T cells from n=4 HIV- donors were activated via CD3/CD28 for 3 days and nucleofected with Dharmacon On target smart siRNA pools specific for RORC2 or a non-targeting (NT) siRNA using the Amaxa technology. RORC2 mRNA expression was quantified by nested real-time PCR (upper panel), while IL-17A production was measured by ELISA (bottom panel; relative production *versus* the NT siRNA control)), at day 3 post-infection.

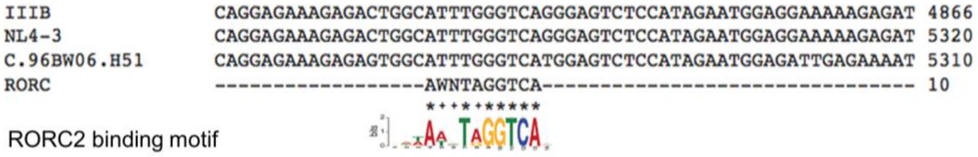

Supporting information Figure 7. RORC2 DNA consensus sequence in HIV-1 Pol. The consensus sequence (Ciofani et al. 2012, Xiao et al. 2014) was aligned using ClustalW2 to the proviral genome sequence of HIV-1 IIIB, NL4.3 and subtype C 96BW06.H51.

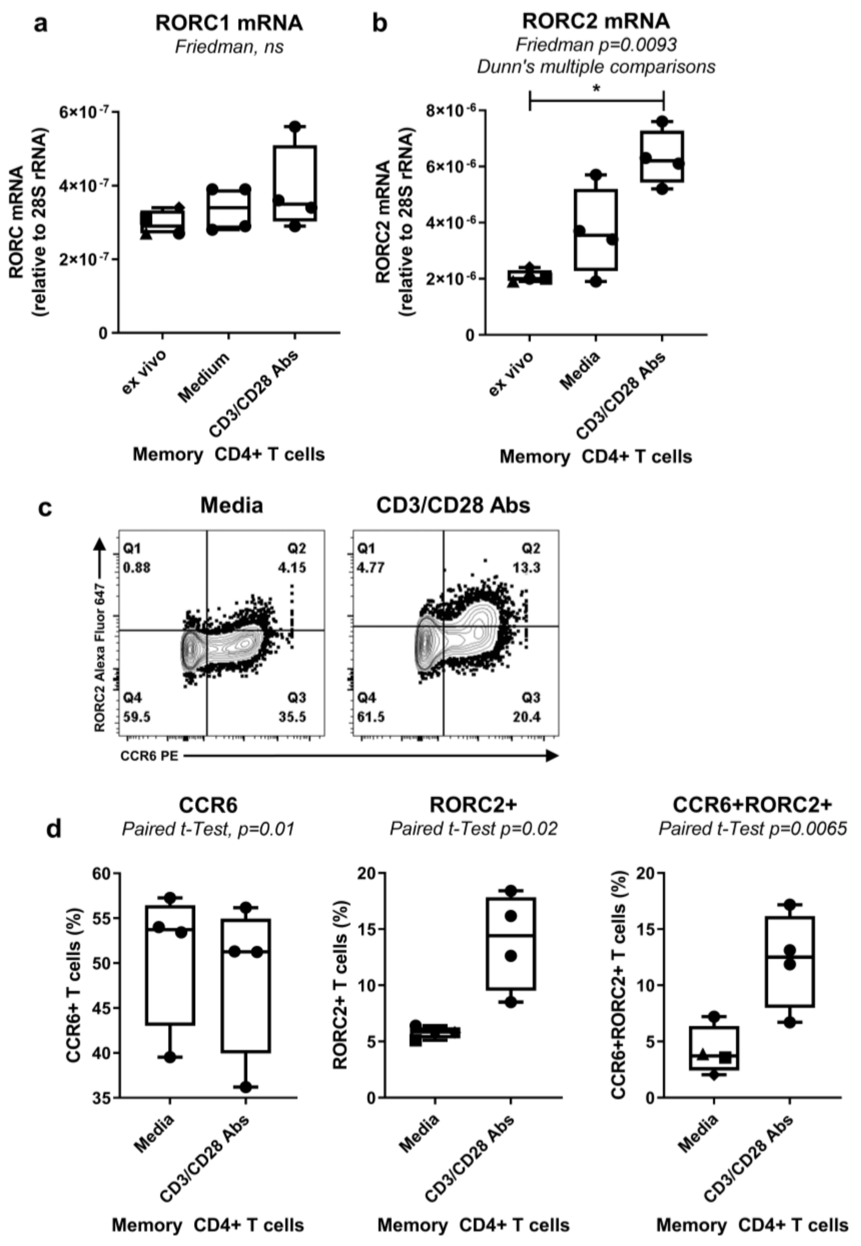

Supporting information Figure 8. TCR triggering promotes RORC2 expression in primary CD4⁺ T cells without interfering with CCR6 expression. Memory CD4⁺ T cells isolated from n=4 HIV- uninfected individuals were stimulated with CD3/CD28 Abs for 5 hours to evaluate RORC1 and RORC2 mRNA expression by real-time RT-PCR and for 24 hours to evaluate RORC2 and CCR6 expression by flow cytometry. Shown are the statistical analysis of RORC1 **(a)** and RORC2 mRNA **(b)** expression *ex vivo*, and in CD3/CD28-activated and non-activated (medium) T cells. Gating strategy and frequency of cells expressing RORC2 and/or CCR6 in one representative individual **(c)** and statistical analysis of the frequency of CCR6+, RORC2+ and CCR6+RORC2+ cells in n=4 individuals **(d)**.

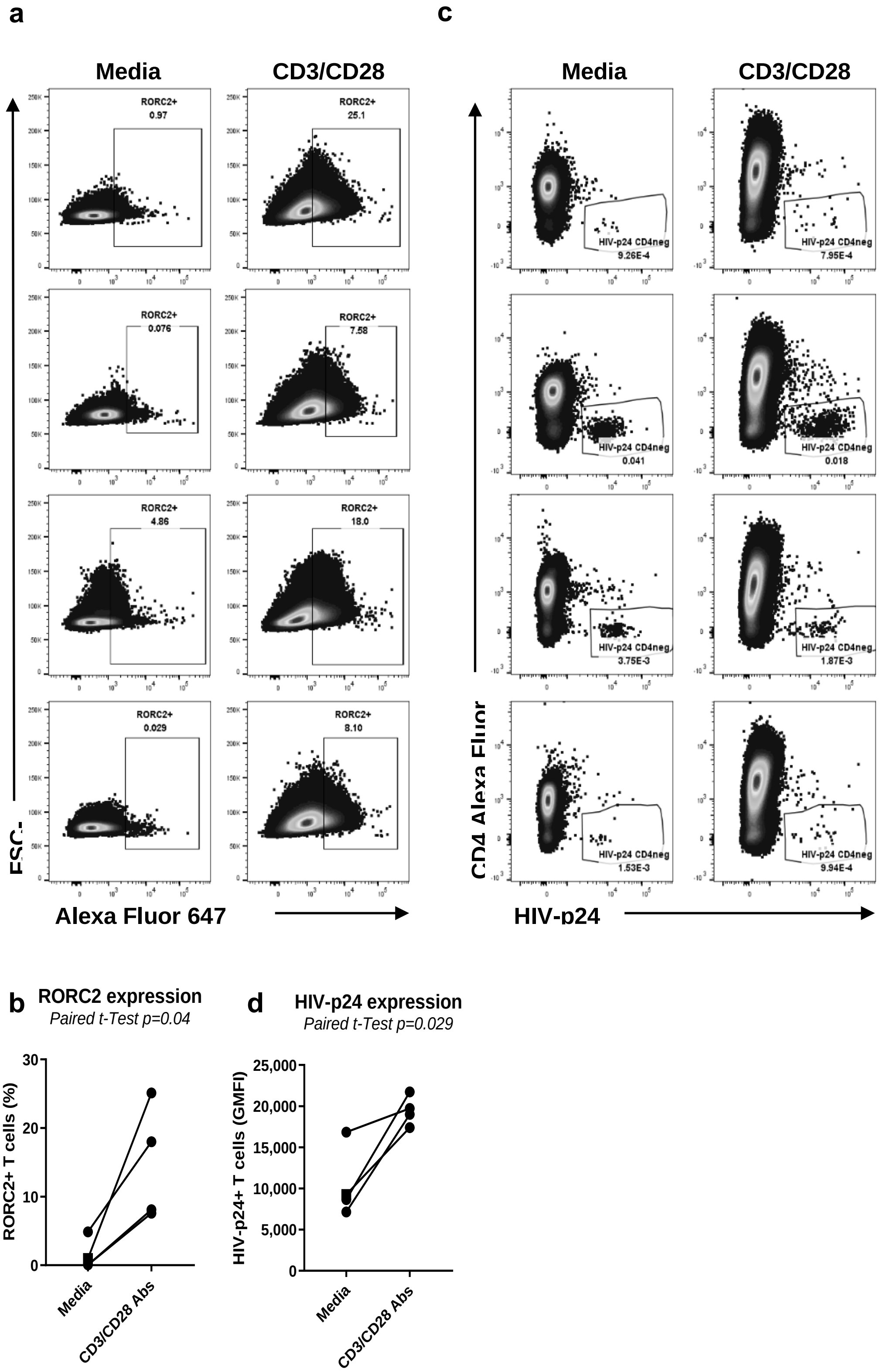

Supporting information Figure 9. Inducible expression of RORC2 and HIV-p24 after TCR triggering. Memory CD4+ T cells isolated from n=4 ART- PLWH individuals were stimulated or not with CD3/CD28 Abs for 3 days. Intracellular expression of RORC2 and HIV-p24 by flow cytometry upon staining with fluorochrome-conjugated CD3, CD4, RORC2 and HIV-p24 Abs. Contour plots for RORC2 expression on cells stimulated (right panels) or not (left panel) with CD3/ CD28 Abs for 3 days **(a)**. Statistical analysis of the frequency of RORC2+ T cells in n=4 individuals **(b)**. Contour plots for CD4^{low}HIV-p24^{+} T cells after stimulation with (right panels) or without (left panel) CD3/ CD28 Abs for 3 days **(c)**. The geometric meanfluorescence intensity (GMFI) of HIV-p24 expression in within CD4^{low}HIV-p24^{+} T cells **(d)**. Paired t-Test values are indicated on the graphs.

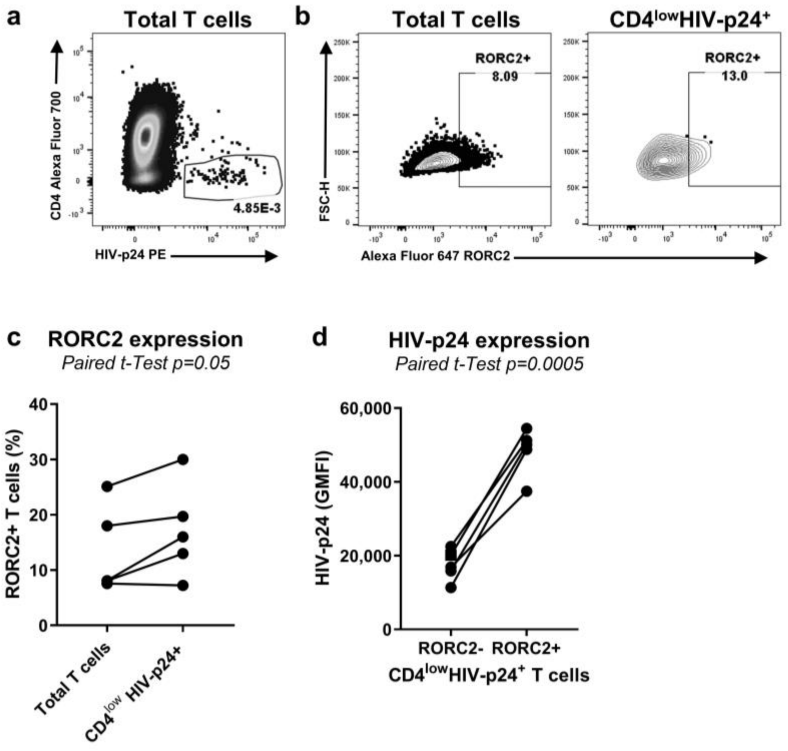

Supporting information Figure 10. High RORC2 expression in memory CD4$^+$ T cells of ART-PLWH carrying translationally-competent HIV reservoirs. Memory CD4+ T cells isolated from n=5ART- PLWH individuals were stimulated with CD3/CD28 Abs for 3 days in the presence of antiretroviral drugs (raltegravir 0.2 µM and BMS806 1 µM) to prevent HIV cell-to-cell transmission *in vitro*. Intracellular expression of HIV-p24 and RORC2 was quantified by flow cytometry after staining with fluorochrome-conjugated CD3, CD4, RORC2 and HIV-p24 Abs. Contour plots for CD4 and HIV-p24 expression, with CD4^{low}HIV-p24$^+$ T cells identified as productively infected cells **(a)**. RORC2 expression in total T cells (CD3+) and CD4^{low}HIV-p24$^+$ T cells in one representative individual **(b)**. Statistical analysis of the frequency of RORC2$^+$ T cells n=5 individuals **(c)** and the geometric meanfluorescence intensity (GMFI) of HIV-p24 expression in RORC2- and RORC2+ within CD4^{low}HIV-p24$^+$ T cells **(d)**. Paired t-Test values are indicated on the graphs.

Supplementary Materials and Methods

Synthesis of GSK GSK2691805A

^{1}H NMR spectral data were recorded on a Bruker 400 NMR spectrometer operating at 400 MHz. $CDCl_3$ is deuterochloroform, DMSO-d6 is hexadeuterodimethyl sulfoxide. Chemical shifts are given in parts per million (δ) downfield from the NMR solvent. Abbreviations for NMR data are as follows: s = singlet, d = doublet, t = triplet, q = quartet, m = multiplet, dd = doublet of doublets, dt = doublet of triplets, app = apparent, br = broad. Step 1: To a solution of ethyl 2-(4-ethylsulfanylphenyl) acetate (1g, 4.7mmol) in dichloromethane (50mL) at 0°C was added 3-chloroperbenzoic acid (2.4g, 14.0mmol) and the reaction was stirred for 16 h at room temperature. The reaction was then quenched with saturated aqueous sodium carbonate solution (50mL) and extracted into dichloromethane (2 x 30mL). The organics were separated, dried ($MgSO_4$) and reduced *in vacuo*. Purification on the Biotage gave ethyl 2-(4-ethylsulfonylphenyl) acetate (980mg, 81% yield) as a gum. ^{1}H NMR ($CDCl_3$): δH 1.25-1.30 (m, 6H), 3.11 (q, 2H), 3.71 (s, 2H), 4.18 (q, 2H), 7.49 (d, 2H) and 7.86 (d, 2H).

Step 2: To a solution of ethyl 2-(4-ethylsulfonylphenyl) acetate (900mg, 3.5mmol) in ethanol (10mL) was added a solution of sodium hydroxide (562mg, 14mmol) in water (10mL) and the reaction stirred at room temperature for 16h. The ethanol was then reduced in vacuo and the remaining aqueous solution was extracted with dichloromethane (2 x 30mL) and then acidified with 2M aqueous HCl (to pH 1). This solution was then extracted with ethyl acetate (2 x 30mL) and the combined organics were dried ($MgSO_4$) and reduced *in vacuo* to give 2-(4-ethylsulfonylphenyl) acetic acid (710mg, 88% yield). 1H NMR ($CDCl_3$): δH 1.28 (t, 3H), 3.12 (q, 2H), 3.77 (s, 2H), 7.51 (s, 2H) and 7.88 (s, 2H).

Step 3: To a solution of 2-(4-ethylsulfonylphenyl)acetic acid (700mg, 3.1mmol) in dichloromethane (20mL) was added N-(3-dimethylaminopropyl)-N-ethylcarbodiimide hydrochloride (705mg, 3.7mmol), 1-hydroxybenzotriazole hydrate (497mg, 3.7mmol) and 4-bromo-3,5-dichloroaniline (733mg, 3.1mmol) and the reaction was stirred at room temperature for 16h. The solution was then diluted with water (20mL) and extracted into dichloromethane (2 x 20mL). The organics were washed with 2M aqueous HCl solution (30mL), then saturated aqueous $NaHCO_3$ solution (30mL) and brine (30mL), dried ($MgSO_4$), reduced *in vacuo* and purified on the Biotage Isolera to give N-(4-bromo-3,5-dichloro-phenyl)-2-(4-ethylsulfonylphenyl)acetamide (520mg, 37% yield) as a light brown solid. 1H NMR (DMSO): δH 1.10 (t, 3H), 3.27 (q, 2H), 3.84 (s, 2H), 7.60 (d, 2H), 7.84-7.86 (m, 4H) and 10.64 (s, 1H). Step 4: A mixture of N-(4-bromo-3,5-dichloro-phenyl)-2-(4-ethylsulfonylphenyl)acetamide (100mg, 0.22mmol) , 2-(trifluoromethoxy)benzeneboronic acid (91mg, 0.4mmol), tetrakis(triphenylphosphine)palladium(0) (26mg, 0.02mmol) and potassium carbonate (61mg, 0.44mmol) in DMF (2mL) were reacted in the microwave at 100°C for 20min. After cooling to room temperature, the mixture was diluted with ethyl acetate (10mL) and washed with water (10mL). The organics were separated, dried, reduced *in vacuo* and purified on the Biotage Isolera to give N-[3,5-dichloro-4-[2-(trifluoromethoxy)phenyl]phenyl]-2-(4-ethylsulfonylphenyl)acetamide GSK2691805A (25mg, 21% yield) as an off-white solid. 1H NMR ($CDCl_3$): δH 1.32 (t, 3H), 3.15 (q, 2H), 3.84 (s, 2H), 7.24-7.27 (m, 1H), 7.36-7.42 (m, 3H), 7.46-7.50 (m, 1H), 7.55 (d, 2H), 7.66 (s, 2H) and 7.91 (d, 2H).

Homogenous Time Resolved Fluorescence (HTRF) RORC2 Ligand Binding Assay

The Homogenous Time Resolved Fluorescence (HTRF) RORC2 Ligand Binding assay measures the interaction of co-factor SRC1 peptide with a purified bacterial-expressed RORγ ligand-binding domain (LBD). This assay is based on the knowledge that nuclear hormone

receptors interact with cofactors in a ligand dependent manner. RORγ has a basal level of interaction with the co-activator SRC1 in the absence of ligand, thus it is possible to find ligands that enhance or inhibit the RORγ/SRC1 peptide interaction. The sites of interaction have been mapped to LXXLL Leucine Charge Domain 2 motifs in the co-factor sequence, and to the AF2 domain of the nuclear receptor. Short peptide sequences containing the LXXLL motif mimic the behaviour of full-length co-factors. A biotinylated SRC1 peptide residues 676-700 (CPSSHSSLTERHKILHRLLQEGSPS-CONH2) was used as an inhibitor (i.e. 'cold peptide') to compete-off the biotinylated peptide from the RORγ LBD. The biotinylated peptide can be displaced by the unbiotinylated form. For this assay, an equal volume of biotinylated SRC1 peptide/Europium-labeled streptavidin (Perkin Elmer) was added to biotynlated RORγ /APC-labeled strepavidin (Perkin Elmer), each in 10 mM DL-dithiothreotol (DTT, JT Baker) with 400 nM D-biotin (Pierce), to give a final solution of 20 nM biotinylated-SRC1 peptide, 1 nM Europium-streptavidin, 20 nM biotinylated-RORγ, 10 nM APC-streptavidin in 10 mM DTT with 400 nM biotin. After a 5 minute incubation at room temperature, 25 µL of the peptide/RORγ solution was added to 384-well assay plates containing 1 µL of titrations of compounds in 100% DMSO. Plates were incubated for one hour and read on ViewLux ultra HTS Microplate Imager (Perkin Elmer) in Lance mode for EU/APC. For dose response curves the APC counts (@671) were divided by the europium (@618) counts to compensate for quenching effects and account for well to well variation due to liquid handling errors. Data were normalized using the following equation: (unknown- ave basal)/ (ave basal - ave background) * 100 = % activation or inhibition. A response of 0% would be inactive, >0% indicates enhancement of the protein peptide interaction and < 0% (negative values) indicates inhibition of the protein peptide interaction. Results were analyzed with ActivityBase (IDBS) using a 4-parameter fit equation.

Jurkat KD RORC2

To generate 1G5 Jurkat indicator cells with a stable KD of RORC2, four shRNA hairpins were obtained from Sigma mission catalogue 3-1245h1C1; 4-1036h21C1; 4-363h21C1 and 3-978h1C1 and were cloned into lentiviral vector pLKO.1-puro (Addgene, catalogue #8543). Lentiviral vectors were produced in 293T cells by Fugene transfection and the supernatant was used to infect Jurkat cells. Forty-eight hours post-infection, cells were selected with 5 µM puromycin for 4 days.

Jurkat RORC2 IL-17F Promoter Luciferase Reporter Assay

The Jurkat RORC2 IL-17F Promoter Luciferase Reporter Assay measures RORC2-specific (human IL-17 conserved non-coding sequence (CNS)) promoter driven luciferase-reporter activity to indirectly assess RORC2 activity. Both RORC gene and reporter construct were sequentially transfected into Jurkat cells and stably integrated. The Jurkat double stable cell line expressing the human RORC2 and IL-17 luciferase-reporter construct was adjusted to a concentration of 0.5 x 10^6 cells/mL in medium (RPMI-1640, 10% FBS, 2 mM Glutamax) and mixed with 166 ng/mL of anti-CD3 antibody (GlaxoSmithKline). 50 µL of cells/antibody was added to each well of 384-well plates containing compound titrations in 100% DMSO and plates were incubated at 37 °C for 18 h. 20 µL of Steady-Glo Luciferase Assay reagent (Promega) was added to each well and plates were incubated for 30 minutes at room temperature. Luminescence was measured on a ViewLux ultra HTS Microplate Imager (Perkin Elmer) and data were analyzed with ActivityBase (IDBS) fitting a 4-parameter equation.

CFSE-based proliferation assay

A modified short-term VOA was performed,as described in Figure 7A. Briefly, memory $CD4^+$ T cells of ART+PLWH were loaded with Carboxy Fluoroscein Succinimidyl Ester (CFSE) (0.5 μM CFSE; Sigma) for 8 minutes at room temperature. The CFSE reaction was then rapidly stopped with human serum for 1-2 minutes. Cells were subsequently washed once with PBS and once with RPMI1640 before being cultured at 1×10^6 cells/well in 1 ml of proliferation media (RPMI, 10% human serum, 1% HEPES, 1% L-glutamine, 1% Penicillin/Streptomycin). The VOA was stopped at day 5 post-activation at which point cells were harvested, stained with Live/Dead Fixable Aqua Dead Cell Stain Kit (Vivid, Life Technologies, Burlington, Ontario, CA) to exclude dead cells and analysed with the BD LSRFortessa and FlowJo version 10 (Tree Star, Inc., Ashland Oregon, USA). Division and proliferation index were calculated using the proliferation tool of Flow Jo. The Division index represents the average number of cell divisions that a cell in the original population has undergone and the Proliferation index represents the total number of divisions divided by the number of cells of cells that went into division.

Western blot

$2x10^6$ cells were centrifugated (100g, 5minutes), washed with ice cold phosphate buffered saline (PBS) once and lysed with 150μl of SDS sample buffer (140mM Tris pH8, 2% SDS, 50 mM DTT, 0.5M sucrose, 2mM $MgCl_2$, Bromophenol-blue). Lysates were incubated at 100°C for 5 minutes. Samples were loaded to a NuPAGE™ Bis-Tris Protein Gel. Western blotting was performed following wet electrophoretic transfer (1h, 100mV) to a PVDF membrane. After probing with the primary antibody at 4^0C overnight, HRP conjugated secondary antibodies were used for detection by luminescence. Primary antibodies were: rabbit polyclonal anti-RORC/RORC2 cat. LS-C40832, rabbit polyclonal anti-RORC/RORC2 L-C116877 both from LSBio (Seattle, WA) and mouse mAb anti-c-Myc (ThermoFisher, 9E10).

qPCR

For Taqman qPCR, approximately $1x10^6$ Jurkat cells were washed twice in PBS and total DNA was extracted with the Qiamp® DNA Minikit (Qiagen, Manchester, UK). Quantitative PCR reactions were carried out as previously described (1) using 0.3pmol each primer and 0.15 pmol of the probe in 25 μL volume containing 100-300ng total DNA using an ABI Prism® 7000 Sequence Detection System (SDS). For amplification of (-) DNA strand (GFP), primers used were forward: CAACAGCCACAACGTCTATATCAT, reverse ATGTTGTGGCGG ATCTTGAAG and probe 5'-FAM-CCG ACA AGC AGA AGA ACG GCA TCA A-3'TAMRA. For amplification of 2LTR circular DNA, the same conditions were used with primers 2LTRqPCR-F: 5'-AACTAGAGATCCCTCAGACCCTTTT-3' and 2LTRqPCR-RC: 5'-CTTGTCTTCGTTGGGAGTGAATT-3' and 2LTR probe 5'-FAM-CTAGAGTTTTCCACACTGAC-0-TAMRA-3'. Standards were prepared by PCR amplification of DNA from acutely infected cells with primers 2LTRF 5'-GCCTCAATAAAGCTTGCCTGG-3' and 2LTRRC 5'-TCCCAGGCTCAGATCTGGTCTAAC-3'. The amplification product was cloned into TOPO vector, amplified and confirmed by sequencing (1). Alu-LTR Taqman qPCR was carried out as previously described (1) using primers ALU-forward, AAC TAG GGA ACC CAC TGC TTA AG and LTR1-reverse, TGC TGG GAT TAC AGG CGT GAG (for first round amplification) and ALU-forward AAC TAG GGA ACC CAC TGC TTA AG, LTR2-reverse, TGC TAG AGA TTT TCC ACA CTG ACT, ALU-probe, FAMRA – TAG TGT GTG CCC GTC TGT TGT GTG AC – TAM (for second round Taqman qPCR). Integrated HIV-DNA in human primary memory $CD4^+$ T-cells was quantified by nested real-time PCR using specific primers and amplification conditions as previously described (2, 3).

RT-qPCR

Jurkat cells were grown in RPMI + 10% FCS. 1.5 x 10^6 cells/ml were seeded onto 12-well plates. Cells were infected with HIV-1 LAI_{GFP} and 24 hours cells an aliquot was analyzed by FACS and another aliquot was treated with GSK2837269A (5 µM) or DMSO. After 24 hours, cells were collected, and RNA was isolated using RNAesy mini kit (Qiagen) and reverse transcribed using SuperScript™ III Reverse Transcriptase (Invitrogen) after digestion with RNAse-free DNAse I (Promega). Quantitative TaqMan qPCR was performed using GFP forward primer (5'-CAA CAG CCA CAA CGT CTA TAT CAT-3'), GFP reverse primer (5'-ATG TTG TGG CGG ATC TTG AAG-3') and GFP probe (5'-FAM-CCG ACAAGC AGA AGA ACG GCA TCA A- TAM-3') in an Eppendorf MasterCycler Realplex. HIV-GFP mRNA expression was normalized to that of hu28S rRNA expression using hu28S rRNA forward primer (5'-TTG AAA ATC CGG GGG AGA G-3') and hu28S rRNA reverse primer (5'-ACA TTG TTC CAA CAT GCC AG-3'). For GFP mRNA, the TaqMan PCR assay was set in a final volume of 20µl containing 250ng cDNA, 0.5µM of each primer, 0.15µM of GFP probe and TaqMan universal master mix II (2X) (Qiagen). For hu28S rRNA, the SYBR Green qPCR reaction was carried out in a final volume of 20µl using 250 ng cDNA, 0.4 µM hu28S rRNA forward and reverse primers and PowerUp™ SYBRTM Green Master Mix. For primary memory $CD4^+$ T-cells, Jurkat, ACH2, TZM-bl and HT-29 cell lines shown in **Supporting information Figure 1**, total RNA was extracted using the RNeasy kit (Qiagen) and quantified by Pearl nanophotometer (Implen, Germany). RORC1 and RORC2 gene expression was evaluated by One step SYBR green real-rime RT-PCR (Qiagen) using a Light-Cycler 480 II as follows; reverse transcription at 50°C for 30 min, 15 min at 95°C and then 45 cycles each at 94°C for 10 s, 61°C for 10 s, and 72°C for 10 s. The sequence of primers used for RORC isoforms were: RORC2 rRNA forward primer, 5'--CTGCTGAGAAGGACAGGGAG-3'; RORC1 rRNA forward primer, 5'-CACAGAGACAGCACCGAGC-3'; RORC2/RORC1 rRNA reverse primer (same for both isoforms) 5'-AGTTCTGCTGACGGGTGC-3'. The relative expression of RORC was normalized relative to the internal control 28S. The sequence of primers used for 28S were 28s rRNA forward primer 5′-CGAGATTCCTGTCCCCACTA-3′ and 28s rRNA reverse primer, 5′ GGGGCCACCTCCTTATTCTA-3′. Primers were obtained from IDT. Each reaction was performed in triplicates. After real time amplification, melting curve analysis was used to determine the uniformity of the thermal dissociation profile for each amplification product.

RNA interference in primary CD4+ T cells

RNA interference was performed as previously described by our group (4). Briefly, PBMCs were thawed and rested overnight at 37 °C. Memory CD4+ T-cells were isolated by negative selection using magnetic beads (Miltenyi Biotec), as described previously (2, 3). Cells were stimulated by CD3/CD28 Abs for 2 days and nuclofected with 100 µM RORC or non-targeting (NT1) siRNA (ON-TARGETplus SMART pool, Dharmacon) using the Amaxa Human T cell Nucleofector Kit (Amaxa, Lonza), according to the manufacturer's protocol. Cells were suspended in the NF solution (100 µl/2×106 cells) and nucleofected using the Amaxa Nucleofector II Device and the human activated T-cell protocol (T-20). Cells (2×106) were transferred into 48-well plates containing 1 ml of RPMI1640 (10 % FBS, 5 ng/ml IL-2, w/o antibiotics) and cultured for another 24 hours at 37 °C before HIV exposure.

ELISA

HIV-p24 levels in cell culture supernatant were quantified using a homemade sandwich ELISA, as described previously (3, 5). Briefly, virions in cell supernatants were lysed using a

homemade buffer solution (PBS 1X, Tween 20 0.05%, Triton X-100 2.5%, Trypan Blue 1% and Thimerosal 0.02% in deionized water) for 1h at 37°C. Levels of IL-17A and IFN-γ were measured in the cell culture supernatant, according to the manufacturer's protocols (Thermofisher).

Supporting references

1. Vozzolo L*, et al.* (2010) Gyrase B inhibitor impairs HIV-1 replication by targeting Hsp90 and the capsid protein. *J Biol Chem* 285(50):39314-39328.
2. Wacleche VS*, et al.* (2016) New insights into the heterogeneity of Th17 subsets contributing to HIV-1 persistence during antiretroviral therapy. *Retrovirology* 13(1):59.
3. Planas D*, et al.* (2017) HIV-1 selectively targets gut-homing CCR6+CD4+ T cells via mTOR-dependent mechanisms. *JCI Insight* 2(15).
4. Cleret-Buhot A*, et al.* (2015) Identification of novel HIV-1 dependency factors in primary CCR4(+)CCR6(+)Th17 cells via a genome-wide transcriptional approach. *Retrovirology* 12:102.
5. Gosselin A*, et al.* (2017) HIV persists in CCR6+CD4+ T cells from colon and blood during antiretroviral therapy. *Aids* 31(1):35-48.
